# Early assessment, diagnosis and treatment of Parkinsonism and Related Syndromes study (ExPRESS): a protocol for an observational study on incident parkinsonism

**DOI:** 10.1136/bmjopen-2026-123017

**Published:** 2026-07-29

**Authors:** Riona Giulia Fumi, Isabella Caraiscos, Edwin Jabbari, Timothy Rittman, James B Rowe, Yoav Ben-Shlomo, Michele T Hu, Huw R Morris

**Affiliations:** 1 Department of Clinical and Movement Neurosciences, University College London, London, UK; 2 Movement Disorders Centre, University College London, London, UK; 3 Department of Clinical Neurosciences and MRC Cognition and Brain Sciences Unit, University of Cambridge, Cambridge, UK; 4 Population Health Sciences, University of Bristol, Bristol, UK; 5 Nuffield Department of Clinical Neurosciences, University of Oxford, Oxford, UK

**Keywords:** Parkinson-s disease, Neurology, Wearable Devices

## Abstract

**Introduction:**

Existing evidence from cohort studies of atypical parkinsonism has demonstrated that median time from symptom onset to diagnosis is 3.4 years for progressive supranuclear palsy (PSP) and 3.1 years for multiple system atrophy (MSA). This compares to only 1.0–1.2 years in Parkinson’s disease (PD). Earlier diagnosis is essential to facilitate recruitment to disease-modifying treatment trials. In the Early Assessment, Diagnosis and Treatment of Parkinsonism and Related Syndromes study, we will prospectively assess patients across the UK with incident parkinsonism and determine the best predictors to improve early diagnosis of the ‘Parkinson-plus’ syndromes (PPS) and build a trial-ready cohort.

**Methods and analysis:**

In this longitudinal prospective clinical cohort study, we will recruit 500 people with incident parkinsonism or syndromes associated with later PPS. Participants must be within 12 months of their first secondary care appointment for either motor disturbance or other symptoms of prodromes to PPS. Participants will return a self-completed questionnaire about their symptoms and have a structured neurological assessment by a clinician capturing key clinical features, clinical diagnostic criteria and quantitative clinical scales. Blood for DNA and plasma will be collected at baseline. 120 participants recruited to a subset of study sites will be invited to undergo detailed biomarker assessments such as additional blood collection, lumbar puncture, skin biopsy, MRI, cognitive assessments and digital biomarker assessments.

We will stratify participants according to their most likely clinical diagnosis at 4 years post-baseline as the gold standard clinical diagnosis. This will be supplemented by neuropathological examination where available. We will use multivariable logistic modelling to identify early predictors of atypical PPS versus PD. Lifetime follow-up will be carried out using medical records linkage with participant consent.

**Ethics and dissemination:**

Ethical approvals have been granted by the Queen Square Research Ethics Committee (Ref: 14/LO/1575), with approval from the Health Research Authority and each participating site. Findings from the study will be published in academic journals and presented at UK-wide and international conferences, as well as disseminated via patient advocacy groups and charities including the PSP Association, Cure PSP, Rare Dementia Support and the MSA Trust.

**Strengths and limitations of this study**

- This is a large inception study of parkinsonism, meaning we will recruit participants from a very early disease stage. This will enable collection of multimodal data relating to early and/or potential prediagnostic cases to enable more accurate earlier diagnosis.
- We will recruit participants with both early typical and atypical forms of parkinsonism.
- Participants will be followed up longitudinally and will extend beyond study visit completion via clinical record linkage. End-point diagnoses will be validated with final clinical diagnosis or pathological follow-up where available.
- Recruitment of early-stage participants from secondary care may pose challenges given clinical care waiting times.

## Introduction

Parkinson’s disease (PD) is the most common cause of parkinsonism, with a prevalence of approximately 138 per 100 000 worldwide and an increasing global burden of disease.^1^ It is characterised by core symptoms of bradykinesia, rigidity and tremor, which are commonly managed with levodopa medication. However, there are many alternative causes to parkinsonism. These include typical presentations of progressive supranuclear palsy (PSP), corticobasal syndrome (CBS) and multiple system atrophy (MSA), some forms of frontotemporal dementia (FTD), and intermediate phenotypes with cognitive and motor features. Approximately half of people presenting with the non-fluent variant primary progressive aphasia (nfvPPA) type of FTD progress in 3 years to a clinical phenotype resembling PSP or CBS. Together, PSP, CBS and MSA are often known as Parkinson-plus syndromes (PPS), and are clinically and pathologically quite distinct from PD.^2^

PSP is caused by a 4-repeat (4R) tauopathy and presents with varying combinations of ocular motor dysfunction, postural instability, akinesia and cognitive dysfunction.^3^ Most epidemiological studies of PSP have demonstrated an estimated prevalence of between 3 and 7 per 100 000 based on the classical form of PSP, PSP-Richardson’s syndrome, using the National Institute of Neurological Disorders and Stroke-PSP criteria.^4–8^ Takigawa *et al* reported a higher estimated prevalence of 17.5 per 100 000 in a large Japanese study.^9^

CBS is a clinically and pathologically heterogeneous syndrome characterised by asymmetric akinetic-rigidity, dystonia, myoclonus, apraxia, cortico-sensory deficit and alien limb phenomena.^10^ Though often caused by the 4R-tauopathy known as corticobasal degeneration (CBD), CBS can also be caused by PSP and Alzheimer’s disease (AD) pathologies, as well as by other pathologies such as TDP-43 in rarer cases.^11 12^ Its estimated prevalence ranges from 2 to 4 per 100 000.^4–7^

MSA is caused by an oligodendroglial and neuronal alpha-synucleinopathy which has distinct seeding properties to that seen in PD-type Lewy body pathology.^13^ Its clinical presentation is primarily characterised by parkinsonism, prominent autonomic dysfunction and/or a cerebellar syndrome.^14^ Reported prevalence of MSA ranges from 0.5 to 17 per 100 000.^15 16^

The PPS are often initially misdiagnosed with PD, despite distinctive clinical features (eg, symmetry, tremor, language, apathy, treatment response, early falls).^17^ In case–control studies of PSP, PD is reported as an initial clinical diagnosis in up to 55% of cases.^18–20^ Swallow and Counsell showed that in a combined group of PSP and CBS cases in the Parkinsonism Incidence in North-East study, 53.6% received an initial diagnosis of PD.^21^ People who are initially misdiagnosed with PD are likely to be rediagnosed within the first 2 years of a PD diagnosis, as shown by a critical evaluation of the stability of a PD diagnosis across four medical institutions in Finland.^22^ Here, rediagnoses from PD included PSP, MSA and clinically undetermined parkinsonism (CUPS). Rediagnosis from PD to CUPS following expert review heavily suggests that the clinical diagnostic criteria for PD are often not stringently applied in a healthcare setting, which is a crucial contributor to diagnostic delay of the PPS.

Initial misdiagnoses of the PPS are not restricted to PD, and may include other conditions such as dementia with Lewy bodies (DLB), frontotemporal dementia (FTD), AD, Creutzfeldt-Jakob disease, myelopathy, myasthenia gravis, stroke, functional neurological disorder and pure autonomic failure (PAF).^21 23–25^ Clinicopathological studies assessing the accuracy of diagnosis of PD, PSP, CBS and MSA have shown that misdiagnoses are common, whereby the positive predictive value (PPV) of the PPS ranges between 80% and 90%.^26–28^ The most common misdiagnosis of PSP, CBS and MSA is PD, followed by other PPS and other pathologies such as DLB, AD and frontotemporal lobar dementia.^27^ A recent clinicopathological study showed that up to 40% of patients with pathologically confirmed PSP had no parkinsonism at first clinical presentation^29^ which may drive the misdiagnosis of early PSP with cognitively presenting disorders.

The initial misdiagnosis of the PPS with PD and other non-PPS syndromes significantly contributes to a lengthy delay between symptom onset and clinical diagnosis. Epidemiological studies have shown that the delay between symptom onset and diagnosis in the PPS ranges from 1.3 years to 3.3 years.^20 24 30–32^ In MSA, those with non-motor symptom onset experience a significantly longer delay to clinical diagnosis compared with those with motor onset, with a delay of up to 4.8±2 years in non-motor onset.^25^ In the PROSPECT study, a longitudinal UK cohort of atypical parkinsonism, we have shown that median time from symptom onset to diagnosis was 3.4 and 3.1 years, respectively, versus an average diagnostic delay of 1.0–1.2 years in PD as per the reported literature.^33 34^

Possible reasons for delayed diagnosis include delay to acknowledgement of index symptoms, especially cognitive and language features of early PSP and CBS resembling nfvPPA or FTD, delays in seeking a primary care appointment, lack of awareness of discriminating features (‘red flags’) in primary care or even secondary care leading to inaccurate diagnoses of PD, secondary care referral waiting times and poor application of the clinical diagnostic criteria in clinical settings. The Movement Disorders Society (MDS) 2017 diagnostic criteria for oligosymptomatic or prodromal PSP have shown good sensitivity to subsequent Richardson’s syndrome.^35^ Although patient-dependent factors such as delays in acknowledgement of symptoms and delays in seeking a healthcare appointment influence diagnostic delay in PSP and CBS, overall diagnostic delay is most impacted after contact with a healthcare professional.^30^

Though revisions to the PSP and MSA clinical diagnostic criteria have included ‘suggestive of’ and ‘possible prodromal’ diagnostic categories to identify patients at the earliest disease stages,^3 14^ diagnostic delay remains a key challenge. It has critical implications on directing patients to specialist clinical care as well as referrals to specialist charities such as the PSP Association and the MSA Trust, which help patients and families to plan both logistically and emotionally for progression to death within 5–10 years.^36^ Qualitative research by Respondek *et al* has shown that diagnostic delay plays a large part in the emotionally taxing journey of ‘cycling through the healthcare system’.^37^ Furthermore, early diagnosis is important for allocation to trials for disease-modifying treatments which have stringent eligibility criteria in line with a therapeutic window.

## Objectives

### Primary objective

In the Early Assessment, Diagnosis and Treatment of Parkinsonism and Related Syndromes (ExPRESS) study, we will recruit 500 people with incident parkinsonism and/or related PPS prodromes such as progressive non-fluent aphasia and PAF between October 2024 and July 2030. The primary aim of this study is to identify clinical, genetic, protein, imaging and digital biomarker features associated with early PSP, CBS and MSA within 12 months of a patient’s first relevant secondary care appointment. Using a multimodal approach, we aim to identify the best early predictors of PD and the PPS to supplement validated clinical diagnostic criteria.

### Secondary objectives

1. To assess the diagnostic delay in PD versus the PPS.
2. To understand the diagnostic pathway in PD and the PPS.
3. To identify baseline features predictive of rediagnosis from PD to a PPS.
4. To assess how the stringent application of clinical diagnostic criteria for PD, PSP, CBS/CBD, MSA and DLB can improve diagnostic accuracy with and without additional research measures.
5. To describe progression to disability in PD versus PPS, and its predictors.

## Methods and analysis

### ExPRESS network

**Figure 1:**
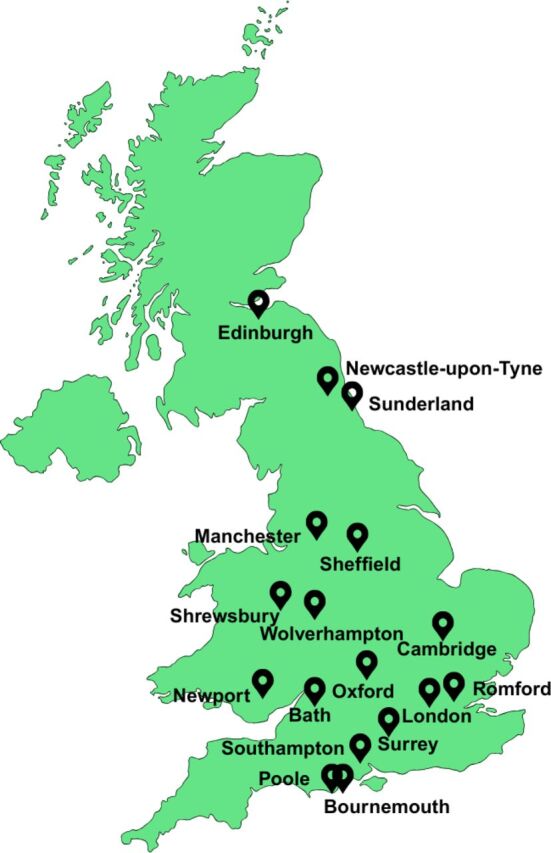
Participating sites across England, Scotland and Wales in the ExPRESS study. ExPRESS, Early Assessment, Diagnosis and Treatment of Parkinsonism and Related Syndromes.

The ExPRESS network is a multicentre UK-wide network currently comprising study sites with clinical or research expertise in parkinsonism. The network is led by Professor Huw Morris (University College London (UCL)), Professor James Rowe (University of Cambridge), Dr Timothy Rittman (University of Cambridge), Professor Michele Hu (University of Oxford) and Professor Yoav Ben-Shlomo (University of Bristol). There are currently 18 centres across England, Scotland and Wales participating in the ExPRESS study (figure 1). Further sites are being added to increase the geographic and ancestry-based diversity. The ExPRESS study network is embedded within the larger PROSPECT network, which includes participants with atypical parkinsonism of all durations.

### Patient and public involvement

In collaboration with the PSPA, we consulted patients and carers about study purpose, design and dissemination plans for the ExPRESS study via support groups and focus groups, which were run both online and in-person. In these sessions, we explained the purpose and outline of the ExPRESS study and invited feedback from both patients and carers. We also consulted the PSPA Research Involvement Members group about study design. We subsequently designed patient questionnaires to be as concise as possible in line with advice about not making questionnaires too long and time-consuming.

### Recruitment

Eligibility criteria state that participants must be within 12 months of their first relevant secondary care appointment for parkinsonism or a related prodrome such as speech and language impairment in nfvPPA (including aphasia and/or PPA) or autonomic dysfunction in PAF. Participants may have been given a pre-existing diagnosis of PD, PSP, CBS, MSA, DLB, indeterminate parkinsonism, a related prodrome or may have no current diagnosis. Participants must be aged 18 or over. Participants with another neurological or psychiatric illness which would significantly impact study participation will be excluded. We will recruit eligible participants from neurology, cognitive or elderly care clinics, or other secondary care services. Participants may self-refer to the study team directly or through patient charities such as Parkinson’s UK, the PSPA or the MSA Trust via ethically approved advertisements on the respective charity websites. We will ask study sites to aim to recruit an approximate minimum of 5 participants per year, however the recruitment of as many participants as possible based on local capacity will be encouraged, and we have not imposed minimum or maximum recruitment numbers.

People with both PD-like or atypical parkinsonism will be recruited to the ExPRESS study. We aim to recruit participants at an approximate 50:50 ratio where approximately half are deemed by the clinician to have a ≥80% likelihood of PD based on medical records and/or referral letters, and half have a <80% likelihood of PD (ie, likely atypical) to enhance statistical power. We aim to recruit 500 incident parkinsonism cases. Assuming 25% (125) of all participants turn out to have a PPS diagnosis, we will have a 3:1 ratio of PD to PPS and can detect a 0.5 year difference between PD and PPS with 82% power, assuming 5% alpha and after excluding 50 subjects with the longest 10% of delays (80% PPS and 20% PD), which will skew the means. If there are a larger number of PPS cases, then our power will be further enhanced.

### Study design

**Figure 2:**
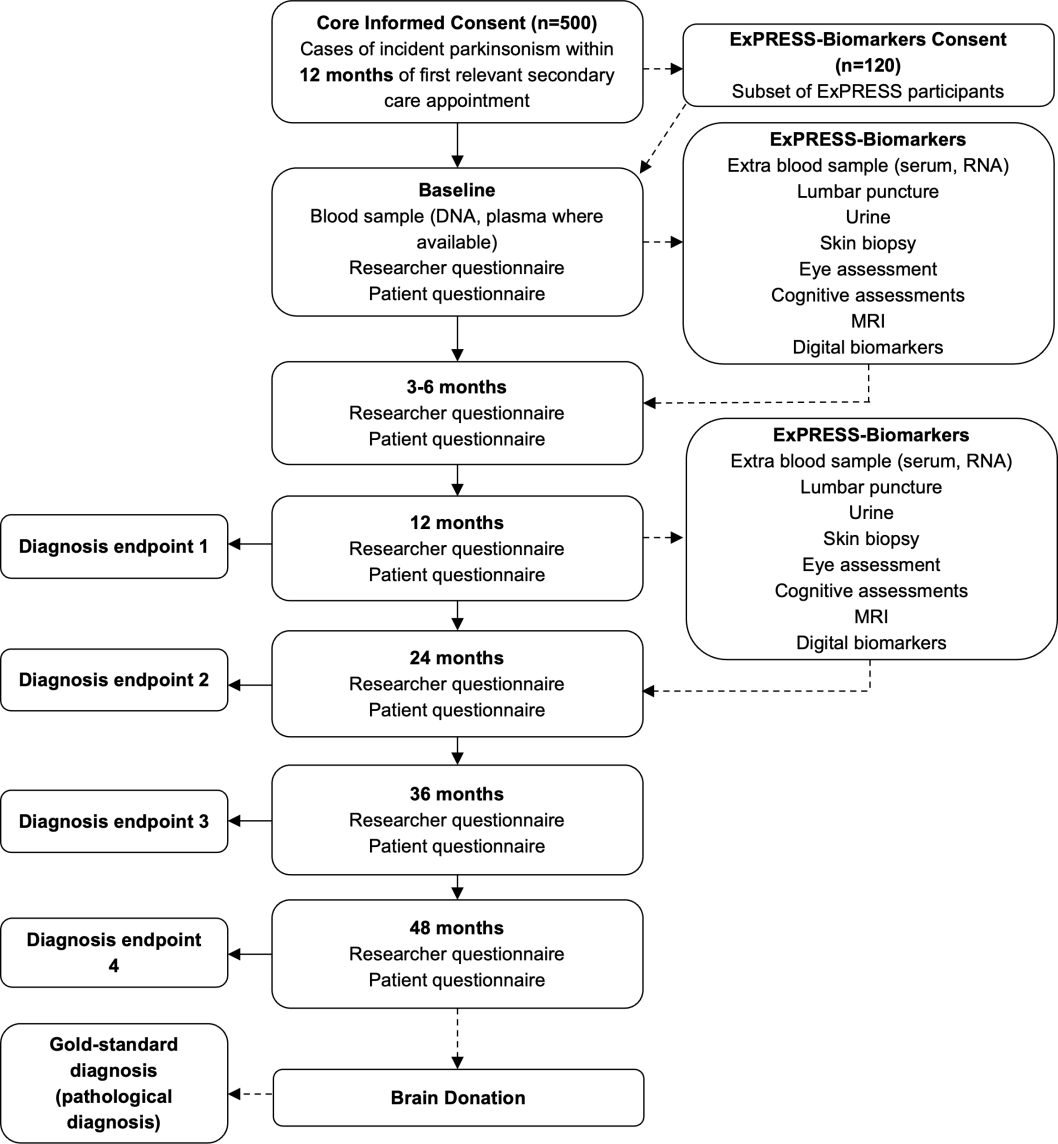
Study assessments within the ExPRESS study. n refers to the targeted number of recruits by the end of 2030. Dotted lines denote optional procedures. ExPRESS, Early Assessment, Diagnosis and Treatment of Parkinsonism and Related Syndromes.

This is a prospective clinical inception cohort study. Study visits will take place at baseline, 6 months, 12 months, 24 months, 36 months and 48 months (see figure 2). At baseline, participants will be asked to donate a blood sample for DNA and a blood sample for plasma at sites with capacity to process and store samples locally.

Each study site will have delegated clinicians (neurologists, geriatricians or other specialists who are referred patients for autonomic or cognitive disorders) and clinical researchers (research nurses or research assistants) to complete study assessments. At each study visit, a researcher questionnaire will be completed, either following a routine clinical appointment or during a dedicated research visit. Neurological assessment will be conducted by a clinician and the questionnaire may be completed by the same clinician or by a delegated clinical researcher based on notes from the neurological examination. The following clinical features are captured within the researcher questionnaire at baseline: falls, urinary incontinence, behavioural change, visual hallucinations, rapid eye movement sleep behaviour disorder, levodopa treatment, levodopa treatment response, intersecting pentagons, verbal fluency in 1 min, vertical saccade impairment, rigidity, speech impairment, PD and PSP-type bradykinesia, dyspraxia, tremor, resting tremor, jerky movements, dystonia, spasticity, asymmetry, posture, gait freezing, lying or sitting blood pressure and standing or lying blood pressure after 3 min. We will ask clinicians to provide the most likely clinical diagnosis at the end of assessment and will be asked to complete checklists for the clinical diagnostic criteria where possible.^3 10 14 38 39^ We will also collect clinical diagnostic certainty level and second or differential diagnosis. Further sections of the researcher questionnaire include the Clinical Impression of Severity Index for Parkinson’s Disease scale (CISI-PD) and the Schwab and England Activities of Daily Living scale (SEADL).

The researcher questionnaire will be repeated at all follow-up visits. In the follow-up researcher questionnaires, we will ask further questions dedicated to clinical test results, including MRI scan, bladder ultrasound, lumbar puncture, PET scan and caeruloplasmin test results. The MDS-Unified Parkinson’s Disease Rating Scale (MDS-UPDRS) Part Ia and the Montreal Cognitive Assessment (MoCA) are also included within the follow-up questionnaires. Should participants be unable to attend an in-person appointment, a video or telephone call will be arranged to collect as much information as possible.

Participants will also be asked to complete questionnaires at each time point. Patient questionnaires will capture basic demographic information, family history, patient history including time and nature of onset, most prominent symptom, asymmetry, key clinical features (falls, urinary dysfunction, visuospatial deficit), levodopa medication and levodopa response. Patients will also be asked to complete the MDS-UPDRS parts Ib and II and the MDS-Cortical Basal Ganglia Functional Scale. Participants will be able to complete questionnaires via an online link, or on paper where they will be posted to the study site.

A subset of 120 participants will be invited to donate extra biomarkers at baseline and 12 months as part of the *ExPRESS-Biomarkers* study at UCL, King’s College London, Cambridge, Oxford, Manchester and Sunderland sites. Participants will be invited to donate extra blood samples, cerebrospinal fluid (CSF), urine and a skin biopsy. They will also undergo extra assessments such as a cognitive examination comprising the MoCA, Addenbrooke’s Cognitive Examination (ACE-III), the Edinburgh Cognitive and Behavioural ALS Screen (ECAS) and a neuropsychometry battery, which have been shown to provide key phenotypic signals in the PPS.^40^ Finally, those in the *ExPRESS-Biomarkers* study will have an MRI scan (based on the QMINMC protocol; https://www.rittman.uk/qminmc/), an eye assessment and digital biomarker assessments as developed by the Oxford Parkinson’s Disease Centre (OPDC).^41^

The alpha-synuclein seed amplification assay (SAA) and high throughput proteomic analysis, for example, NUcleic acid Linked Immuno-Sandwich Assay (NULISA)-CNS-220 will be carried out on plasma and CSF to define biomarkers that can distinguish between PPS and PD in early-stage patients. Further fluid biomarkers will be analysed as they become available.

Digital biomarker assessments will consist of an 8-minute smartphone test performed in clinic assessing gait, balance, tremor, dexterity, reaction time and voice, as developed by the OPDC and previously evaluated across PD, prodromal PD and control participants.^42^ Smartphone-based assessments can reliably detect parkinsonism, offering a scalable, objective approach to separating PPS from PD that remains underexplored.

Oculomotor tests will comprise a 10-minute battery, including fixation, horizontal and vertical gaze, smooth pursuit, pro-saccades, anti-saccades, self-paced saccades and sympathetic/parasympathetic function on pupillometry. Each of these has been shown to be abnormal in some PPS, but have not been compared directly across diagnoses.^43 44^

Participants will be asked for consent to view their clinical summary records via the NHS National Care Record Service and will be asked for consent for their general practitioner (GP) or consultants to share their medical records, enabling routine remote follow-up beyond study completion.

### Storage of clinical data

Study sites will enter collected data into a Research Electronic Data Capture (REDCap), a secure web-based software platform. The database is restricted to authorised study site team members through role-based logins. For MRI scans, a separate imaging platform (XNAT) is used. Digital biomarker data are uploaded directly to the KneuHealth platform (secure cloud infrastructure managed via Amazon Web Services best practices), via the OPDC smartphone application.

### Storage of biomaterials

EDTA Blood samples for DNA extraction will be sent to the Rare Inherited Disorders Genomic Laboratory in London. All other biosamples (plasma, serum, CSF, urine and skin biopsies) will be processed in line with the protocol of the Parkinson’s Progression Markers Initiative (PPMI)^45^ and stored locally at −70°C. Samples will be transferred to central storage at the Institute of Neurology, UCL, on a periodic basis.

### Brain banking

At the time of consent, participants will be asked whether they would like to receive information on brain donation. Brain banking is not centralised and is performed at the following individual centres: Queen Square Brain Bank, Oxford Brain Bank, Cambridge Brain Bank, Newcastle Brain Tissue Resource or Imperial Brain Bank depending on geographical location. Should participants consent to receiving information, they will be provided with information and consent forms or referred directly to the brain bank by the ExPRESS study team.

At the time of death, confirmation of tissue retrieval will be provided by brain bank coordinators. Postmortem reports will be shared with the central study team as per the participant’s consent to provide pathological verification and allow for clinicopathological analyses.

### Analysis plan

We will aim to identify and validate baseline clinical, genetic, protein, imaging and digital biomarker features which are predictive of final diagnosis. Unless participants are withdrawn before completion of the study, the most recent diagnosis end-point if not pathological diagnosis will be diagnosis end-point 4 (48 months post-baseline) (see figure 2). For prediction of final (end-point 4) diagnosis, we will use multivariable logistic regression modelling and decision-tree based machine learning algorithms (such as XGBoost) to identify key clinical features, quantitative scale markers (such as total and sub-score from the MDS-UPDRS, CBFS and MoCA), genetic variants (such as LRRK2, TRIM11, ApoE status and tau haplotype), SAA status and protein biomarker levels (such as neurofilament light, tau and amyloid-beta markers defined from NULISA) collected at baseline which could significantly predict a diagnosis of PD, PSP, CBS or MSA.

With regard to secondary study aims, we will assess the diagnostic delay in PD and each PPS with reference to dates of symptom onset (motor and/or cognitive and/or autonomic) and final diagnosis (end-point 4 diagnosis), and compare diagnostic delay between groups. We will review the diagnostic pathway for each end-point diagnosis by collating data from prospectively collected patient and researcher questionnaires, as well as retrospective review of medical records to extract data preceding study enrolment. For predicting rediagnosis in patients who are given an initial diagnosis (end-point diagnosis 1) of PD, we will perform similar multivariable logistic regression modelling and decision-tree based machine learning algorithms as described above. We will apply separate models for those in whom the PD diagnosis was retrospectively appropriate or not appropriate (contrasting phenotypic evolution vs misdiagnosis).

Further, we will assess diagnostic accuracy in parkinsonism syndromes using methods as previously described in clinico-pathological research.^26^ We will compare pathological diagnosis with each end-point diagnosis and calculate the PPV and sensitivity of each clinical diagnosis. We will group cases into the following pathology groups based on primary pathological diagnosis: Lewy Body Disease (LBD), PSP, CBD, AD with clinical presentation of CBS (CBS-AD), MSA and ‘other’. We will classify true positives according to the following criteria: LBD pathology with a final clinical diagnosis of PD or DLB, PSP pathology with a final clinical diagnosis of PSP or CBS, CBD pathology with a clinical diagnosis of CBS, AD pathology with a final clinical diagnosis of CBS and MSA pathology with a final clinical diagnosis of MSA.

We will perform exploratory analysis to assess how application of the clinical diagnostic criteria in the researcher questionnaire impacts diagnostic accuracy, which will be completed dependent on time availability in the research assessment. Finally, we will use Cox proportional hazard regression for modelling clinical progression to disability across different disease groups, using a score of <40% in the SEADL or a score of >4 in the CISI-PD disability classification as an indication of disability. We will assess how the emergence of clinical features, change in research scale scores and biomarker levels over time points impact progression to death or disability within each end-point diagnosis group.

For all analyses, data on each clinical feature will be brought together from various different sources. Where both questionnaires have been completed, researcher-reported data will be prioritised over patient-reported data to code clinical features, with the aim of extracting data with the highest accuracy.

The MRI images will be used to assess changes in relation to diagnosis, phenotype, prognosis and progression. These changes will be examined from T1w (regional changes in volumes and thickness), T2w (white matter hyperintensities), diffusion-weighted (tractography and tract-based diffusion metrics) iron-sensitive imaging (nigrosomal and brain stem nuclei/loci) and task-free functional MRI (functional connectivity and derived metrics).

We will assess digital smartphone features derived from the 8 min in clinic assessment and compare these features between different PPS groups to identify whether they can improve the accuracy of early diagnosis. Smartphone-based machine-learning classifiers to (1) detect parkinsonism versus healthy controls and (2) differentiate APS from PD will be developed and evaluated using smartphone feature extraction, SHAP-based feature selection within stratified groups, k-fold cross-validation and trained XGBoost classifiers. Composite scores based on top-performing smartphone features will also be generated to separate PD from APS, and PSP/CBD from MSA cases.

Oculomotor testing will produce quantified parameters from reflexive and volitional saccades, anti-saccades and smooth pursuit including latency, velocity, error rate and gain that will be compared across the APS groups using previously published methods.^38 39^

## Discussion

This longitudinal prospective clinical cohort study of parkinsonism aims to help with the early differential diagnosis between PD and other degenerative causes of parkinsonism including PPS and FTD/PPA prodromes. Similar prospective longitudinal studies in early PD patients such as the CamPaIGN study,^46^ PPMI,^45^ Tracking Parkinson’s^47^ and OPDC Discovery Cohort study^48^ have provided crucial insight into the progression of PD, but have been restricted to the recruitment of participants with a clinical diagnosis of PD, with early cases defined as 3 and 3.5 years from onset in OPDC and Tracking Parkinson’s, respectively. Conversely, epidemiological and convenience cohort studies of PPS and FTD have lacked matched PD cases, such as the PiPPIN study (Coyle Gilchrist 2016, Murley 2020) and the PROSPECT study.^17 49^ We propose that by recruiting participants at a 50:50 ratio of ≥80% and <80% likelihood of PD, respectively, our cohort will comprise a group of PPS participants with the most relevant disease-control group of typical PD.

We anticipate that the eligibility criteria of 12 months from the first relevant secondary care appointment may pose challenges. Several study sites are tertiary care centres, where patients may have already attended several appointments prior to specialist referral. The predominant participant referral source for the ExPRESS study will be from neurology clinics run by movement disorders consultants and cognitive disorders specialists within the pre-existing PROSPECT network. Patients who attend their first secondary care appointment in a non-neurology clinic may therefore not be identified quickly enough for study recruitment. The high proportion of neurology-centred ExPRESS sites imposes limitations on participant recruitment in those with prodromal features, who are of particular interest and value in identifying truly prodromal cases. This may be offset by the higher index of suspicion for differential diagnoses in specialist centres, and more confident, accurate non-PD diagnoses.

Baseline publications of UK-wide observational parkinsonism cohorts show that a disproportionately high proportion of participants with white European ancestry are typically recruited to Parkinson’s research. Cohorts from the PPMI, Tracking Parkinson’s and OPDC studies are made up of 95%–97% white participants.^47 50 51^ To mitigate this risk, we aim to build a representative cohort and have boosted recruitment or initiated site set-up in areas with a high population of people with non-European ancestry, for example, London North West University Healthcare NHS Trust, King’s College Hospital NHS Foundation Trust and the Whittington Health NHS Trust.

The option for remote questionnaire completion and consent to access patient clinical records will serve a critical purpose in allowing data collection remotely and providing an alternative means of assessment should participants become too impaired to visit a study site. Without remote follow-up, drop-out from in-person study visits leads to a bias in less severely impaired disease presentations. It is important to note that this bias is not entirely mitigated by the option for remote follow-up, as completion of full study scales such as the MDS-UPDRS remains a challenge at this stage. The addition of eye movement recording provides a simple non-invasive metric that could ultimately provide a quick readout in clinic. The option for remote testing is intended to improve retention and reduce participant burden, although we recognise the challenges of a ‘digital divide’ in the UK population and sites will seek to mitigate this in discussion with potential participants.

The primary long-term beneficiaries of this study will be patients. Surveys of those with lived experience consistently hear of the demand for an earlier and more accurate diagnosis by quicker referral to specialist care. In a focus group about the ExPRESS study, we asked patients and carers about their perceived benefits of improving early diagnosis. The feedback highlighted that receiving an earlier diagnosis would have reduced anxiety levels and would’ve given families more time to prepare for the future. Additionally, earlier diagnosis facilitates earlier engagement with supporting charities and more options for clinical trials.

We hope that the ExPRESS study will also raise awareness of the differential diagnoses for PD, including PPS, among patients, carers and healthcare professionals, particularly those working in primary care. A survey run by the PSPA in 2023 showed that some patients saw their GP up to five times before being referred to a neurologist, representing lack of awareness and contributing to delay to diagnosis.^52^

## Ethics and dissemination

Ethical approvals have been granted by the Queen Square Research Ethics Committee, as part of the wider PROSPECT study (Ref: 14/LO/1575). Participants will provide informed consent and a study partner will be optionally assigned. A consultee process is in place should participants lack capacity to consent, though we expect the grand majority of participants to have capacity at the specified eligibility window. Capacity will be assessed at each follow-up visit and the study partner will be asked to sign a consultee form should this be required.

Findings from the ExPRESS study will be published in academic journals and presented at UK-wide and international conferences, and communicated by collaborating charities such as the PSP Association, Cure PSP, Rare Dementia Support and the MSA Trust. Patients, carers and members of the public will be invited to attend meetings and research days showcasing the results of the ExPRESS study, often in collaboration with charity research days. We will continue to involve patients and carers in both study design and results at charity-led support groups.

## Supplementary Material

Reviewer comments

Author's manuscript
