# Supplementary material for: Early assessment, diagnosis and treatment of Parkinsonism and Related Syndromes study (ExPRESS): a protocol for an observational study on incident parkinsonism
Source: BMJ Open. 2026 Jul 29;16(7):e123017. doi: 10.1136/bmjopen-2026-123017 (PMC13479499; doi:10.1136/bmjopen-2026-123017)
Supplement: Supplementary file 1 [file bmjopen-2026-123017.draft_revisions.pdf]

BMJ Open is committed to open peer review. As part of this commitment we make the peer review history of every article we publish publicly available.

When an article is published we post the peer reviewers' comments and the authors' responses online. We also post the versions of the paper that were used during peer review. These are the versions that the peer review comments apply to.

The versions of the paper that follow are the versions that were submitted during the peer review process. They are not the versions of record or the final published versions. They should not be cited or distributed as the published version of this manuscript.

BMJ Open is an open access journal and the full, final, typeset and author-corrected version of record of the manuscript is available on our site with no access controls, subscription charges or pay-per-view fees (http://bmjopen.bmj.com).

If you have any questions on BMJ Open's open peer review process please email info.bmjopen@bmj.com

BMJ Open

The Early assessment, diagnosis and treatment of Parkinsonism and Related Syndromes study (ExPRESS): a protocol for an observational study on incident parkinsonism

Journal:	*BMJ Open*
Manuscript ID	bmjopen-2026-123017
Article Type:	Protocol
Date Submitted by the Author:	21-May-2026
Complete List of Authors:	Fumi, Riona; University College London, Department of Clinical and Movement Neurosciences; University College London, Movement Disorders Centre<br>Caraiscos, Isabella ; University College London, Department of Clinical and Movement Neurosciences; University College London, Movement Disorders Centre<br>Jabbari, Edwin ; University College London, Department of Clinical and Movement Neurosciences; University College London, Movement Disorders Centre<br>Rittman, Timothy; University of Cambridge, Department of Clinical Neurosciences and MRC Cognition and Brain Sciences Unit<br>Rowe, James; University of Cambridge, Department of Clinical Neurosciences and MRC Cognition and Brain Sciences Unit<br>Ben-Shlomo, Yoav; University of Bristol, Population Health Sciences<br>Hu, Michele; University of Oxford, Nuffield Department of Clinical Neurosciences<br>Morris, Huw ; University College London, Department of Clinical and Movement Neurosciences; University College London, Movement Disorders Centre
Keywords:	Parkinson-s disease < NEUROLOGY, Neurology < INTERNAL MEDICINE, Wearable Devices < Wearable Electronic Devices

SCHOLARONE MANUSCRIPTS

The Early assessment, diagnosis and treatment of Parkinsonism and Related Syndromes study (ExPRESS): a protocol for an observational study on incident parkinsonism

Riona G. Fumi, Isabella Caraiscos, Edwin Jabbari, Timothy Rittman, James B. Rowe, Yoav Ben-Shlomo, Michele T. Hu and Huw R. Morris.

Corresponding author: Riona Giulia Fumi; 33 Queen Square, London, WC1N 3BG; E: riona.fumi@ucl.ac.uk; T: 02031082838.

Author contacts: riona.fumi@ucl.ac.uk, i.caraiscos@ucl.ac.uk, e.jabbari@ucl.ac.uk, tr332@medschl.cam.ac.uk, James.Rowe@mrc-cbu.cam.ac.uk, y.ben-shlomo@bristol.ac.uk, michele.hu@ndcn.ox.ac.uk, h.morris@ucl.ac.uk.

Key words: progressive supranuclear palsy, corticobasal syndrome, multiple system atrophy, Parkinson's disease

Word count: 4046

For peer review only

ABSTRACT

Introduction

Existing evidence from the PROSPECT-M-UK study of atypical parkinsonism has demonstrated that median time from symptom onset to diagnosis is 3.4 years for progressive supranuclear palsy (PSP) and 3.1 years for multiple system atrophy (MSA). This compares to only 1.0-1.2 years in Parkinson's disease (PD). Earlier diagnosis is essential to facilitate recruitment to disease-modifying treatment trials. In the Early Assessment, Diagnosis and Treatment of Parkinsonism and Related Syndromes study (ExPRESS), we will prospectively assess patients across the UK with incident parkinsonism and determine the best predictors to improve early diagnosis of the "Parkinson-plus" syndromes (PPS) and build a trial-ready cohort.

Methods and analysis

We will recruit 500 people with incident parkinsonism or syndromes associated with later PPS. Participants must be within 12 months of their first secondary care appointment for either motor disturbance or other symptoms of prodromes to PPS. Participants will return a self-completed questionnaire about their symptoms and have structured neurological assessment capturing key clinical features, clinical diagnostic criteria and quantitative clinical scales. Blood for DNA and plasma will be collected at baseline. A subset of 120 participants will be invited to undergo detailed biomarker assessments such as additional blood collection, lumbar puncture, skin biopsy, magnetic resonance imaging (MRI), cognitive assessments and digital biomarker assessments.

We will classify participants according to their most likely clinical diagnosis at 4 years post-baseline as the gold standard clinical diagnosis. This will be supplemented by neuropathological examination where available. We will use multivariable logistic modeling to identify early predictors of atypical PPS versus Parkinson's disease (PD). Long term follow-up will be carried out using NHS medical records linkage with participant consent.

Ethics and Dissemination

Ethical approvals have been granted by the Queen Square Research Ethics Committee (Ref: 14/0371), with approval from the Health Research Authority and each participating site. Findings from the study will be published in academic journals and presented at UK-wide and international conferences, as well as dissemination via patient advocacy groups and charities including the PSP Association, Cure PSP, Rare Dementia Support, and the MSA Trust.

ARTICLE SUMMARY

Strengths and limitations of this study

- This is a large inception study of parkinsonism which will capture multi-modal data relating to early and/or potential pre-diagnostic cases to enable more accurate earlier diagnosis
- We will recruit participants with both early typical and atypical forms of parkinsonism

For peer review only - http://bmjopen.bmj.com/site/about/guidelines.xhtml

- Participants will be followed-up longitudinally and will extend beyond study visit completion via clinical record linkage. End-point diagnoses will be validated with final clinical diagnosis or pathological follow-up where available.
- Recruitment of early-stage participants from secondary care may pose challenges given clinical care waiting times.

INTRODUCTION

There are many causes of parkinsonism other than idiopathic Parkinson's disease (PD). These include typical presentations of progressive supranuclear palsy (PSP), corticobasal syndrome (CBS) and Multiple System Atrophy (MSA), some forms of frontotemporal dementia (FTD), and intermediate phenotypes with cognitive and motor features. Approximately half of people presenting with the non-fluent variant primary progressive aphasia type of FTD progress in 3 years to a clinical phenotype resembling PSP or CBS. Together, PSP, CBS and MSA are often known as Parkinson-plus syndromes (PPS), and are clinically and pathologically quite distinct from PD.

PSP is caused by a 4-repeat (4R) tauopathy and presents with varying combinations of ocular motor dysfunction, postural instability, akinesia and cognitive dysfunction (1). Most epidemiological studies of PSP have demonstrated an estimated prevalence of between 3 and 7 per 100,000 based on the classical form of PSP, PSP-Richardson's syndrome, using NINDS-PSP criteria (2–6) Takigawa and colleagues reported a higher estimated prevalence of 17.5 per 100,000 in a large Japanese study (7).

CBS is a clinically and pathologically heterogenous syndrome characterised by asymmetric akinetic-rigidity, dystonia, myoclonus, apraxia, cortico-sensory deficit and alien limb phenomena (8). Though often caused by the 4R-tauopathy known as corticobasal degeneration (CBD), CBS can also be caused by PSP and Alzheimer's disease (AD) pathologies, as well as by other pathologies such as TDP-43 in rarer cases (9,10). Its estimated prevalence ranges from 2 and 4 per 100,000 (2–5).

MSA is caused by an oligodendroglial and neuronal alpha-synucleinopathy which has distinct seeding properties to that seen in PD-type Lewy body pathology (11). Its clinical presentation is primarily characterised by parkinsonism, prominent autonomic dysfunction and/or a cerebellar syndrome (12). Reported prevalence of MSA ranges from 0.5 and 17 per 100,000 (13,14).

The PPS are often initially misdiagnosed with PD, despite distinctive clinical features (e.g. symmetry, tremor, language, apathy, treatment response, early falls) (15). In case-control studies of PSP, PD is reported as an initial clinical diagnosis in up to 55% of cases (16–18). Swallow and Counsell showed that in a combined group of PSP and CBS cases in the PINE study, 53.6% received an initial diagnosis of PD (19). People who are initially misdiagnosed with PD are likely to be re-diagnosed within the first 2 years of a PD diagnosis, as shown by a critical evaluation of the stability of a PD diagnosis across 4 medical institutions in Finland (20). Here, re-diagnoses from PD included PSP, MSA and clinically undetermined parkinsonism (CUPS). Re-diagnosis from PD to CUPS following expert review heavily suggests that the clinical

For peer review only - http://bmjopen.bmj.com/site/about/guidelines.xhtml

diagnostic criteria for PD are often not stringently applied in a healthcare setting, which is a crucial contributor to diagnostic delay of the PPS.

Initial misdiagnoses of the PPS are not restricted to PD, and may include other conditions such as Lewy Body Dementia (LBD), frontotemporal dementia (FTD), AD, Creutzfeldt-Jakob Disease (CJD), myelopathy, myasthenia gravis, stroke, functional neurological disorder and pure autonomic failure (PAF) (19,21–23). Clinico-pathological studies assessing the accuracy of diagnosis of PD, PSP, CBS and MSA have shown that misdiagnoses are common, whereby the positive predictive value of the PPS range between 80 and 90% (24–26). The most common misdiagnosis of PSP, CBS and MSA is PD, followed by other PPS and other pathologies such as DLB, AD and frontotemporal dementia (FTLD) (25). A recent clinico-pathological study showed that up to 40% of patients with pathologically confirmed PSP had no parkinsonism at first clinical presentation (27) which may drive the misdiagnosis of early PSP with cognitively presenting disorders.

The initial misdiagnosis of the PPS with PD and other non-PPS syndromes significantly contributes to a lengthy delay between symptom onset and clinical diagnosis. Epidemiological studies have shown that the delay between symptom onset and diagnosis in the PPS ranges from 1.3 years to 3.3 years (18,22,28–30). In MSA, those with non-motor symptom onset experience a significantly longer delay to clinical diagnosis compared to those with motor onset, with a delay of up to 4.8 ± 2 years in non-motor onset (23). In the PROSPECT-M-UK study, a longitudinal UK cohort of atypical parkinsonism, we have shown that median time from symptom onset to diagnosis was 3.4 and 3.1 years respectively, versus an average diagnostic delay of 1.0-1.2 years in PD as per the reported literature (31,32).

Possible reasons for delayed diagnosis include delay to acknowledgement of index symptoms, especially cognitive and language features of early PSP and CBS resembling nonfluent variant Primary Progressive Apraxia (nfvPPA) or FTD, delays in seeking a primary care appointment, lack of awareness of discriminating features (“red flags”) in primary care or even secondary care leading to inaccurate diagnoses of PD, secondary care referral waiting times and poor application of the clinical diagnostic criteria in clinical settings. The Movement Disorders Society (MDS) 2017 diagnostic criteria for oligosymptomatic or prodromal PSP have shown good sensitivity to subsequent Richardson’s syndrome (33). Although patient-dependent factors such as delays in acknowledgement of symptoms and delays in seeking a healthcare appointment influence diagnostic delay in PSP and CBS, overall diagnostic delay is most impacted after contact with a healthcare professional (28).

Though revisions to the PSP and MSA clinical diagnostic criteria have included ‘suggestive of’ and ‘possible prodromal’ diagnostic categories to identify patients at the earliest disease stages (1,12) diagnostic delay remains a key challenge. It has critical implications on directing patients to specialist clinical care as well as referrals to specialist charities such as the PSP Association and The MSA Trust, which help patients and families to plan both logistically and emotionally for progression to death within 5-10 years (34). Qualitative research by Respondek and colleagues has shown that diagnostic delay plays a large part in the emotionally taxing journey of ‘cycling

For peer review only - http://bmjopen.bmj.com/site/about/guidelines.xhtml

through the healthcare system' (35). Furthermore, early diagnosis is important for allocation to trials for disease-modifying treatments which have stringent eligibility criteria in line with a therapeutic window.

OBJECTIVES

Primary objective

In the ExPRESS study, we will recruit 500 people with incident parkinsonism and/or related PPS prodromes such as progressive non-fluent aphasia (PNFA) and PAF between October 2024 and July 2030. The primary aim of this study is to identify clinical, genetic, protein, imaging and digital biomarker features associated with early PSP, CBS and MSA within 12 months of a patient's first relevant secondary care appointment. Using a multimodal approach, we aim to build an algorithm for early prediction of PPS to supplement validated clinical diagnostic criteria.

Secondary objectives

1) To assess the diagnostic delay in PD vs the PPS
2) To understand the diagnostic pathway in the PPS
3) To identify baseline features predictive of re-diagnosis from PD to a PPS
4) To assess how the stringent application of clinical diagnostic criteria for PD, PSP, CBS/CBD, MSA and DLB can improve diagnostic accuracy with and without additional research measures
5) To describe progression to disability in PD vs PPS, and its predictors.

METHODS AND ANALYSIS

ExPRESS Network

The ExPRESS network is a multi-centre UK-wide network currently comprising study sites with clinical or research expertise in parkinsonism. The network is led by Professor Huw Morris (University College London), Professor James Rowe (University of Cambridge), Dr Timothy Rittman (University of Cambridge), Michele Hu (University of Oxford) and Professor Yoav Ben-Shlomo (University of Bristol). There are currently 18 centres across England, Scotland and Wales participating in the ExPRESS study (*Figure 1*). Further sites are being added to increase the geographic and ancestry-based diversity. The ExPRESS study network is embedded within the larger PROSPECT-M-UK network, which includes participants with atypical parkinsonism of all durations.

Study Funding

The ExPRESS study is jointly funded by the Medical Research Council (MRC) and the National Institute for Health and care Research (NIHR) (MR/Y008219/1) as part of the Rare Disease Research UK initiative (https://rd-research.org.uk/node/express/). The views expressed are those of the authors and not necessarily those of the NIHR or the MRC. The study is also supported by the PSP Association (PSPA 'Understood'), (https://www.pspassociation.org.uk);

For peer review only - http://bmjopen.bmj.com/site/about/guidelines.xhtml

the MSA Trust (https://www.msatrust.org.uk), centre-specific NIHR Biomedical Research Centres, and philanthropy.

In collaboration with the PSP Association, we consulted patients and carers about study purpose, design, and dissemination plans for the ExPRESS study via support groups.

Recruitment

Eligibility criteria state that participants must be within 12 months of their first relevant secondary care appointment for parkinsonism or a related prodrome such as speech and language impairment in nfvPPA (including aphasia and/or PPA) or autonomic dysfunction in PAF. Participants may have been given a pre-existing diagnosis of PD, PSP, CBS, MSA, DLB, indeterminate parkinsonism, a related prodrome or may have no current diagnosis. Participants must be aged 18 or over. Participants with another neurological or psychiatric illness which would significantly impact study participation will be excluded. We will recruit eligible participants from neurology, cognitive or elderly care clinics, or other secondary care services. Participants may self-refer to the study team directly or through patient charities such as Parkinson's UK, the PSP Association or the MSA Trust via ethically approved advertisements on the respective charity websites.

People with PD-like or atypical parkinsonism will be recruited to the ExPRESS study. We aim to recruit participants at a 50:50 ratio where approximately half are deemed to have a ≥80% likelihood of PD and half have a <80% likelihood of PD (i.e. likely atypical) to enhance statistical power. We aim to recruit 500 incident parkinsonism cases. Assuming 25% (125) of all participants turn out to have a PPS diagnosis, we will have a 3:1 ratio of PD to PPS and can detect a 0.5 year difference between PD and PPS with 82% power, assuming 5% alpha and after excluding 50 subjects with the longest 10% of delays (80% PPS and 20% PD) which will skew the means. If there are a larger number of PPS cases then our power will be further enhanced.

Study design

Study visits will take place at baseline, 6 months, 12 months, 24 months, 36 months and 48 months (see *Figure 2*). At baseline, participants will be asked to donate a blood sample for DNA, and a blood sample for plasma at sites with capacity to process and store samples locally.

Delegated clinicians and/or clinical researchers will complete a researcher questionnaire following a routine clinical appointment or a dedicated research visit. The following clinical features are captured within the researcher questionnaire at baseline: falls, urinary incontinence, behavioural change, visual hallucinations, REM sleep behaviour disorder, levodopa treatment, levodopa treatment response, intersecting pentagons, verbal fluency in 1 minute, vertical saccade impairment, rigidity, speech impairment, PD and PSP-type bradykinesia, dyspraxia, tremor, resting tremor, jerky movements, dystonia, spasticity, asymmetry, posture, gait freezing, lying or sitting blood pressure and standing or lying blood pressure after 3 minutes. We will ask clinicians to provide the most likely clinical diagnosis at the end of assessment and will be asked to complete checklists for the clinical diagnostic criteria

For peer review only

For peer review only - http://bmjopen.bmj.com/site/about/guidelines.xhtml

where possible (1,8,12,36,37). We will also collect clinical diagnostic certainty level and second or differential diagnosis. Further sections of the researcher questionnaire include the Clinical Impression of Severity Index for Parkinson's Disease scale (CISI-PD) and the Schwab and England Activities of Daily Living scale (SEADL).

The researcher questionnaire will be repeated at all follow-up visits. In the follow-up researcher questionnaires, we will ask further questions dedicated to clinical test results, including MRI scan, bladder ultrasound, lumbar puncture, PET scan and caeruloplasmin test results. The MDS-Unified Parkinson's Disease Rating Scale (UPDRS) Part Ia and the Montreal Cognitive Assessment (MoCA) are also included within the follow-up questionnaires. Should participants be unable to attend an in-person appointment, a video or telephone call will be arranged to collect as much information as possible.

Participants will also be asked to complete questionnaires at each time point. Patient questionnaires will capture basic demographic information, family history, patient history including time and nature of onset, most prominent symptom, asymmetry, key clinical features (falls, urinary dysfunction, visuospatial deficit), levodopa medication and levodopa response. Patients will also be asked to complete the MDS-UPDRS parts Ib and II and the MDS-Cortical Basal Ganglia Functional Scale (MDS-CBFS). Participants will be able to complete questionnaires via an online link, or on paper where they will be posted to the study site.

A subset of 120 participants will be invited to donate extra biomarkers at baseline and 12 months as part of the *ExPRESS-Biomarkers* study at University College London (UCL), King's College London (KCL), Cambridge, Oxford, Manchester and Sunderland sites. Participants will be invited to donate extra blood samples, cerebrospinal fluid (CSF), urine and a skin biopsy. They will also undergo extra assessments such as a cognitive examination comprising the MoCA, Addenbrooke's Cognitive Examination (ACE-III), the Edinburgh Cognitive and Behavioural ALS Screen (ECAS) and a neuropsychometry battery, which have been shown to provide key phenotypic signals in the PPS (38). Finally, those in the *ExPRESS-Biomarkers* study will have an MRI scan (based on the QMINMC protocol; https://www.rittman.uk/qminmc/), an eye assessment and digital biomarker assessments as developed by the Oxford Parkinson's Disease Centre (OPDC) (39).

Digital biomarker assessments will consist of an 8-minute smartphone test performed in clinic assessing gait, balance, tremor, dexterity, reaction time and voice, as developed by the OPDC and previously evaluated across PD, prodromal PD and control participants (40). Smartphone-based assessments can reliably detect parkinsonism, offering a scalable, objective approach to separating PPS from PD that remains underexplored.

Oculomotor tests will comprise a 10-minute battery, including fixation, horizontal and vertical gaze, smooth pursuit, pro-saccades, anti-saccades, self-paced saccades, and sympathetic/parasympathetic function on pupillometry. Each of these have been shown to be abnormal in some PPS, but have not been compared directly across diagnoses (41,42).

For peer review only - http://bmjopen.bmj.com/site/about/guidelines.xhtml

Participants will be asked for consent to view their clinical summary records via the NHS National Care Record Service (NCRS) and will be asked for consent for their GP or consultants to share their medical records, enabling routine remote follow-up beyond study completion.

Storage of clinical data

Study sites will enter collected data into a Research Electronic Data Capture (REDCap), a secure web-based software platform. The database is restricted to authorised study site team members through role-based logins. For MRI scans, a separate imaging platform (XNAT) is used. Digital biomarker data are uploaded directly to the KneuHealth platform (secure cloud infrastructure managed via Amazon Web Services best practices), via the OPDC smartphone application.

Storage of biomaterials

EDTA Blood samples for DNA extraction will be sent to the Rare Inherited Disorders Genomic Laboratory (RIDGL) in London. All other biosamples (plasma, serum, CSF, urine and skin biopsies) will be processed in line with the protocol of the Parkinson's Progression Markers Initiative (PPMI) (43) and stored locally at -70 degrees Celsius. Samples will be transferred to central storage at the Institute of Neurology, UCL, on a periodic basis.

Brain banking

At the time of consent, participants will be asked whether they would like to receive information on brain donation. Brain banking is not centralised and is performed at the following individual centres: Queen Square Brain Bank, Oxford Brain Bank, Cambridge Brain Bank, Newcastle Brain Tissue Resource or Imperial Brain Bank depending on geographical location. Should participants consent to receiving information, they will be provided with information and consent forms or referred directly to the brain bank by the ExPRESS study team.

At the time of death, confirmation of tissue retrieval will be provided by brain bank coordinators. Post-mortem reports will be shared with the central study team as per the participant's consent to provide pathological verification and allow for clinico-pathological analyses.

Analysis plan

We will aim to identify and validate baseline clinical, genetic, protein, imaging and digital biomarker features which are predictive of final diagnosis. Unless participants are withdrawn before completion of the study, the most recent diagnosis end-point if not pathological diagnosis will be diagnosis end-point 4 (48 months post baseline) (see *Figure 2*). For prediction of final (end-point 4) diagnosis, we will use multivariable logistic regression modelling and decision-tree based machine learning algorithms to identify key clinical features, quantitative scale markers (such as total and sub-score from the MDS-UPDRS, CBFS and MoCA), genetic variants (such as LRRK2, TRIM11, ApoE status and tau haplotype) and protein biomarker levels (such as neurofilament light, tau and amyloid-beta markers) collected at baseline which could significantly predict a diagnosis of PD, PSP, CBS or MSA.

For peer review only - http://bmjopen.bmj.com/site/about/guidelines.xhtml

With regards to secondary study aims, we will assess the diagnostic delay in PD and each PPS with reference to dates of symptom onset (motor and/or cognitive and/or autonomic) and final diagnosis (end-point 4 diagnosis), and compare diagnostic delay between groups. We will review the diagnostic pathway for each end-point diagnosis by collating data from prospectively collected patient and researcher questionnaires, as well as retrospective review of medical records to extract data preceding study enrolment. For predicting re-diagnosis in patients who are given an initial (end-point diagnosis 1) of PD, we will perform similar multivariable logistic regression modelling and decision-tree based machine learning algorithms as described above. We will apply separate models for those in whom the PD diagnosis was retrospectively appropriate or not appropriate (contrasting phenotypic evolution versus misdiagnosis).

Further, we will assess diagnostic accuracy in parkinsonism syndromes using methods as previously described in clinico-pathological research (24). We will perform exploratory analysis to assess how application of the clinical diagnostic criteria in the researcher questionnaire impacts diagnostic accuracy, which will be completed dependent on time availability in the research assessment. Finally, we will use cox proportional hazard regression for modelling clinical progression to disability across different disease groups, using a score of <40% in the SEADL or a score of >4 in the CISI-PD disability classification as an indication of disability. We will assess how the emergence of clinical features, change in research scale scores and biomarker levels over time points impact progression to death or disability within each end-point diagnosis group.

For all analyses, researcher-reported data will be harmonised with patient-reported data. Researcher-reported data will be prioritised over patient-reported data with the aim of extracting data with the highest accuracy.

The MRI images will be used to assess changes in relation to diagnosis, phenotype, prognosis and progression. These changes will be examined from T1w (regional changes in volumes and thickness), T2w (white matter hyperintensities), diffusion-weighted (tractography and tract-based diffusion metrics) iron-sensitive imaging (nigrosomal and brain stem nuclei/loci) and task-free fMRI (functional connectivity and derived metrics).

We will assess whether digital smartphone features derived from the 8-minute in clinic assessment can improve the accuracy of early diagnosis of APS including PSP, CBD and MSA. Smartphone-based machine-learning classifiers to (1) detect parkinsonism versus healthy controls and (2) differentiate APS from PD will be developed and evaluated using smartphone feature extraction, SHAP-based feature selection within stratified groups, k-fold cross-validation and trained XGBoost classifiers. Composite scores based on top-performing smartphone features will also be generated to separate PD from APS, and PSP/CBD from MSA cases.

Oculomotor testing will produce quantified parameters from reflexive and volitional saccades, anti-saccades and smooth pursuit including latency, velocity, error rate and gain that will be compared across the APS groups using previously published methods (insert ref 38 and 39 here).

For peer review only - http://bmjopen.bmj.com/site/about/guidelines.xhtml

DISCUSSION

This inception cohort of parkinsonism aims to help with the early differential diagnosis between PD and other degenerative causes of parkinsonism including PPS and FTD/PPA prodromes. Similar prospective longitudinal studies in early PD patients such as the CamPaIGN study (44), the Parkinson Progression Marker Initiative (PPMI) (43), Tracking Parkinson's (45) and OPDC Discovery Cohort study (46) have provided crucial insight into the progression of PD, but have been restricted to the recruitment of participants with a clinical diagnosis of PD, with early cases defined as 3 and 3.5 years from onset in OPDC and Tracking Parkinson's respectively. Conversely, epidemiological and convenience cohort studies of PPS and FTD have lacked matched PD cases, such as the PiPPIN study (Coyle Gilchrist 2016, Murley 2020) and the PROSPECT-M-UK study (15,47). We propose that by recruiting participants at a 50:50 ratio of ≥80% and <80% likelihood of PD respectively, our cohort will comprise a group of PPS participants with the most relevant disease-control group of typical PD.

We anticipate that the eligibility criteria of 12 months from the first relevant secondary care appointment may pose challenges. Several study sites are tertiary care centres, where patients may have already attended several appointments prior to specialist referral. The predominant participant referral source for the ExPRESS study will be from neurology clinics run by movement disorders consultants and cognitive disorders specialists within the pre-existing PROSPECT-M-UK network. Patients who attend their first secondary care appointment in a non-neurology clinic may therefore not be identified quickly enough for study recruitment. The high proportion of neurology-centred ExPRESS sites imposes limitations on participant recruitment in those with prodromal features, who are of particular interest and value in identifying truly prodromal cases. This may be offset by the higher index of suspicion for differential diagnoses in specialist centres, and more confident, accurate non-PD diagnoses.

Baseline publications of UK-wide observational parkinsonism cohorts show that a disproportionately high proportion of participants with white European ancestry are typically recruited to Parkinson's research. Cohorts from the PPMI, Tracking Parkinson's and OPDC studies are made up of 95-97% white participants (45,48,49). To mitigate this risk, we aim to build a representative cohort and have boosted recruitment or initiated site set-up in areas with a high population of people with non-European ancestry, e.g. London North West University Healthcare NHS Trust, King's College Hospital NHS Foundation Trust and the Whittington Health NHS Trust.

The option for remote questionnaire completion and consent to access patient clinical records will serve a critical purpose in allowing data collection remotely and providing an alternative means of assessment should participants become too impaired to visit a study site. Without remote follow-up, drop-out from in-person study visits leads to a bias in less severely impaired disease presentations. It is important to note that this bias is not entirely mitigated by the option for remote follow-up, as completion of full study scales such as the MDS-UPDRS remains a challenge at this stage. The addition of eye movement recording provides a simple non-invasive

For peer review only - http://bmjopen.bmj.com/site/about/guidelines.xhtml

metric that could ultimately provide a quick readout in clinic. The option for remote testing is intended to improve retention and reduce participant burden, although we recognise the challenges of a “digital divide” in the UK population and sites will seek to mitigate this in discussion with potential participants.

The primary long-term beneficiaries of this study will be patients. Surveys of those with lived experience consistently hear of the demand for an earlier and more accurate diagnosis by quicker referral to specialist care. This also enables earlier engagement with supporting charities and more options for clinical trials.

We hope that the ExPRESS study will also raise awareness of the differential diagnoses for PD, including PPS, among patients, carers and healthcare professionals, particularly those working in primary care. A survey run by the PSPA in 2023 showed that some patients saw their GP up to 5 times before being referred to a neurologist, representing lack of awareness and contributing to delay to diagnosis (50).

ETHICS AND DISSEMINATION

Ethical approvals have been granted by the Queen Square Research Ethics Committee, as part of the wider PROSPECT-M-UK study (Ref: 14/0371). Participants will provide informed consent and a study partner will be optionally assigned. A consultee process is in place should participants lack capacity to consent, though we expect the grand majority of participants to have capacity at the specified eligibility window. Capacity will be assessed at each follow-up visit and the study partner will be asked to sign a consultee form should this be required.

Findings from the ExPRESS study will be published in academic journals and presented at UK-wide and international conferences, and communicated by collaborating charities such as the PSP Association, Cure PSP, Rare Dementia Support, and the MSA Trust. Patients, carers and members of the public will be invited to attend meetings and research days showcasing the results of the ExPRESS study, often in collaboration with charity research days. We will continue to involve patients and carers in both study design and results at charity-led support groups.

Data availability statement

The ExPRESS study shares a Data Access Committee (DAC) with that for the overarching PROSPECT-M-UK study. The DAC aims to encourage scientific collaboration and maximize the impact of data collection to the wider scientific community. A Data Access Application may be made in order to obtain data and/or samples collected as part of the ExPRESS study, by contacting the study coordinator at prospect@ucl.ac.uk.

REFERENCES

1. Höglinger GU, Respondek G, Stamelou M, Kurz C, Josephs KA, Lang AE, et al. Clinical diagnosis of progressive supranuclear palsy: The movement disorder society criteria. Mov Disord. 2017 June;32(6):853–64.

For peer review only - http://bmjopen.bmj.com/site/about/guidelines.xhtml

2. Swallow DMA, Counsell CE. Prevalence of progressive supranuclear palsy and corticobasal syndrome in Scotland. Neuroepidemiology. 2022 June 2;56(4):291–7.

3. Lyons S, Trépel D, Lynch T, Walsh R, O'Dowd S. The prevalence and incidence of progressive supranuclear palsy and corticobasal syndrome: a systematic review and meta-analysis. J Neurol. 2023 Sept;270(9):4451–65.

4. Swallow DMA, Zheng CS, Counsell CE. Systematic review of prevalence studies of progressive supranuclear palsy and corticobasal syndrome. Mov Disord Clin Pract. 2022 July;9(5):604–13.

5. Coyle-Gilchrist ITS, Dick KM, Patterson K, Vázquez Rodríquez P, Wehmann E, Wilcox A, et al. Prevalence, characteristics, and survival of frontotemporal lobar degeneration syndromes. Neurology. 2016 May 3;86(18):1736–43.

6. Logroscino G, Piccininni M, Graff C, Hardiman O, Ludolph AC, Moreno F, et al. Incidence of Syndromes Associated With Frontotemporal Lobar Degeneration in 9 European Countries. JAMA Neurol. 2023;80(3):279–86.

7. Takigawa H, Kitayama M, Wada-Isoe K, Kowa H, Nakashima K. Prevalence of progressive supranuclear palsy in Yonago: change throughout a decade. Brain Behav. 2016 Dec;6(12):e00557.

8. Armstrong MJ, Litvan I, Lang AE, Bak TH, Bhatia KP, Borroni B, et al. Criteria for the diagnosis of corticobasal degeneration. Neurology. 2013 Jan 29;80(5):496–503.

9. Koga S, Josephs KA, Aiba I, Yoshida M, Dickson DW. Neuropathology and emerging biomarkers in corticobasal syndrome. J Neurol Neurosurg Psychiatry. 2022 June 13;93(9):919–29.

10. Alexander SK, Rittman T, Xuereb JH, Bak TH, Hodges JR, Rowe JB. Validation of the new consensus criteria for the diagnosis of corticobasal degeneration. J Neurol Neurosurg Psychiatry. 2014 Aug;85(8):925–9.

11. Koga S, Sekiya H, Kondru N, Ross OA, Dickson DW. Neuropathology and molecular diagnosis of Synucleinopathies. Mol Neurodegener. 2021 Dec 18;16(1):83.

12. Wenning GK, Stankovic I, Vignatelli L, Fanciulli A, Calandra-Buonaura G, Seppi K, et al. The Movement Disorder Society criteria for the diagnosis of multiple system atrophy. Mov Disord. 2022 June;37(6):1131–48.

13. Kaplan S. Prevalence of multiple system atrophy: A literature review. Rev Neurol (Paris). 2024 May;180(5):438–50.

14. Poewe W, Stankovic I, Halliday G, Meissner WG, Wenning GK, Pellecchia MT, et al. Multiple system atrophy. Nat Rev Dis Primers. 2022 Aug 25;8(1):56.

15. Jabbari E, Holland N, Chelban V, Jones PS, Lamb R, Rawlinson C, et al. Diagnosis across the spectrum of progressive supranuclear palsy and corticobasal syndrome. JAMA Neurol. 2020 Mar 1;77(3):377–87.

For peer review only - http://bmjopen.bmj.com/site/about/guidelines.xhtml

16. Viscidi E, Litvan I, Dam T, Juneja M, Li L, Krzywy H, et al. Clinical Features of Patients With Progressive Supranuclear Palsy in an US Insurance Claims Database. Front Neurol. 2021;12:571800.

17. Kwasny MJ, Oleske DM, Zamudio J, Diegidio R, Höglinger GU. Clinical features observed in general practice associated with the subsequent diagnosis of progressive supranuclear palsy. Front Neurol. 2021 Apr 22;12:637176.

18. Street D, Whiteside D, Rittman T, Rowe JB. Prediagnostic Progressive Supranuclear Palsy - Insights from the UK Biobank. Parkinsonism Relat Disord. 2022;95:59–64.

19. Swallow DMA, Counsell CE. The evolution of diagnosis from symptom onset to death in progressive supranuclear palsy (PSP) and corticobasal degeneration (CBD) compared to Parkinson's disease (PD). J Neurol. 2023 July;270(7):3464–74.

20. Raty V, Kuusimaki T, Majuri J, Vahlberg T, Gardberg M, Noponen T, et al. Stability and Accuracy of a Diagnosis of Parkinson Disease Over 10 Years. Neurology. 2025;104(9):e213499.

21. Shea YF, Shum ACK, Lee SC, Chiu PKC, Leung KS, Kwan YK, et al. Natural clinical course of progressive supranuclear palsy in Chinese patients in Hong Kong. Hong Kong Med J. 2019;25(6):444–52.

22. Morgan JC, Ye X, Mellor JA, Golden KJ, Zamudio J, Chiodo LA, et al. Disease course and treatment patterns in progressive supranuclear palsy: A real-world study. J Neurol Sci. 2021 Feb 15;421(117293):117293.

23. Vichayanrat E, Valerio F, Koay S, De Pablo-Fernandez E, Panicker J, Morris H, et al. Diagnosing premotor multiple system atrophy: Natural history and autonomic testing in an autopsy-confirmed cohort: Natural history and autonomic testing in an autopsy-confirmed cohort. Neurology. 2022 Sept 13;99(11):e1168–77.

24. Hughes AJ, Daniel SE, Ben-Shlomo Y, Lees AJ. The accuracy of diagnosis of parkinsonian syndromes in a specialist movement disorder service. Brain. 2002 Apr;125(Pt 4):861–70.

25. Massey Q, Nihoyannopoulos L, Zeidman P, Warner T, Bhatia K, Gandhi S, et al. Refining the diagnostic accuracy of Parkinsonian disorders using metaphenomic annotation of the clinicopathological literature. NPJ Parkinsons Dis. 2025;11(1):314.

26. Wrigley S, Cullinane PW, Parmera JB, Arca VM, Sifontes Valladares W, Rivera-Sánchez M, et al. Clinical diagnosis of progressive supranuclear palsy (PSP): A clinicopathological comparison of patients with confirmed PSP and clinical mimics. Mov Disord. 2025 Oct;40(10):2116–28.

27. Driver-Dunckley ED, Zhang N, Serrano GE, Dunckley NA, Sue LI, Shill HA, et al. Low clinical sensitivity and unexpectedly high incidence for neuropathologically diagnosed progressive supranuclear palsy. J Neuropathol Exp Neurol. 2023 Apr 20;82(5):438–51.

28. Swallow DMA, Murchie P, Counsell CE. Total Patient Delay: A comparison of patient and clinician/health system delays in the diagnosis of progressive supranuclear palsy and corticobasal syndrome. Mov Disord Clin Pract. 2024 May;11(5):478–87.

For peer review only - http://bmjopen.bmj.com/site/about/guidelines.xhtml

29. Marchand F, Blaise A-S, Defebvre L, Cailliau E, Bombois S, Devos D, et al. Clinical prognostic factors in progressive supranuclear palsy: Implications for clinical trials. J Parkinsons Dis. 2024 Nov;14(8):1652–8.

30. Libri I, Altomare D, Bracca V, Rivolta J, Cantoni V, Mattioli I, et al. Time to diagnosis and its predictors in syndromes associated with frontotemporal Lobar Degeneration. Am J Geriatr Psychiatry. 2024 Aug;32(8):1004–13.

31. Breen DP, Evans JR, Farrell K, Brayne C, Barker RA. Determinants of delayed diagnosis in Parkinson's disease. J Neurol. 2013 Aug 10;260(8):1978–81.

32. Cervantes-Arriaga A, Sarabia-Tapia C, Esquivel-Zapata O, López-Alamillo S, Reséndiz-Henriquez E, Corona T, et al. Pitfalls and caveats in the diagnostic pathway of people with ParkinsonÓ?s disease. Revista Mexicana de Neurociencia [Internet]. 2022 Oct 26 [cited 2026 Mar 17];23(4). Available from: http://dx.doi.org/10.24875/RMN.21000041

33. Grimm M-J, Respondek G, Stamelou M, Arzberger T, Ferguson L, Gelpi E, et al. Clinical conditions 'suggestive of Progressive Supranuclear Palsy'-diagnostic performance. Mov Disord. 2020 Dec;35(12):2301–13.

34. Golbe LI. Early diagnosis of progressive supranuclear palsy: bucking the odds. Neurology. 2008 Nov 25;71(22):1754–5.

35. Respondek G, Breslow D, Amirghiasvand C, Ghosh B, Bergmans B, van Wyk L, et al. The lived experiences of people with progressive supranuclear palsy and their caregivers. Neurol Ther. 2023 Feb;12(1):229–47.

36. Postuma RB, Berg D, Stern M, Poewe W, Olanow CW, Oertel W, et al. MDS clinical diagnostic criteria for Parkinson's disease: MDS-PD Clinical Diagnostic Criteria. Mov Disord. 2015 Oct 1;30(12):1591–601.

37. McKeith IG, Boeve BF, Dickson DW, Halliday G, Taylor J-P, Weintraub D, et al. Diagnosis and management of dementia with Lewy bodies: Fourth consensus report of the DLB Consortium. Neurology. 2017 July 4;89(1):88–100.

38. Hu MT, Coma AQ, Rowe JB, Zerenner T, Church A, Fumi R, et al. Cognitive and neuropsychiatric profiles distinguish atypical parkinsonian syndromes. Brain. 2025 Sept 3;148(9):3239–51.

39. Arora S, Baig F, Lo C, Barber TR, Lawton MA, Zhan A, et al. Smartphone motor testing to distinguish idiopathic REM sleep behavior disorder, controls, and PD. Neurology. 2018 Oct 16;91(16):e1528–38.

40. Gunter KM, Groenewald K, Aubourg T, Lo C, Welch J, Razzaque J, et al. Smartphone-based prediction of dopaminergic deficit in prodromal and manifest Parkinson's disease. NPJ Digit Med. 2025 Dec 1;8(1):783.

41. Kassavetis P, Kaski D, Anderson T, Hallett M. Eye movement disorders in movement disorders. Mov Disord Clin Pract. 2022 Apr 16;9(3):284–95.

42. Sekar A, Panouillères MTN, Kaski D. Detecting abnormal eye movements in patients with neurodegenerative diseases - current insights. Eye Brain. 2024 Apr 9;16:3–16.

For peer review only

43. Marek K, Jennings D, Lasch S, Siderowf A, Tanner C, Simuni T, et al. The Parkinson Progression Marker Initiative (PPMI). Prog Neurobiol. 2011 Dec;95(4):629–35.

44. Williams-Gray CH, Mason SL, Evans JR, Foltynie T, Brayne C, Robbins TW, et al. The CamPaIGN study of Parkinson's disease: 10-year outlook in an incident population-based cohort. J Neurol Neurosurg Psychiatry. 2013 Nov;84(11):1258–64.

45. Malek N, Swallow DMA, Grosset KA, Lawton MA, Marrinan SL, Lehn AC, et al. Tracking Parkinson's: Study design and baseline patient data. J Parkinsons Dis. 2015;5(4):947–59.

46. Szewczyk-Krolikowski K, Tomlinson P, Nithi K, Wade-Martins R, Talbot K, Ben-Shlomo Y, et al. The influence of age and gender on motor and non-motor features of early Parkinson's disease: initial findings from the Oxford Parkinson Disease Center (OPDC) discovery cohort. Parkinsonism Relat Disord. 2014 Jan;20(1):99–105.

47. Street D, Jabbari E, Costantini A, Jones PS, Holland N, Rittman T, et al. Progression of atypical parkinsonian syndromes: PROSPECT-M-UK study implications for clinical trials. Brain. 2023 Aug 1;146(8):3232–42.

48. Sfikas E, Koros C. Association of sporadic and genetic Parkinson's disease with cancer: Insights from the PPMI cohort. Genes (Basel). 2025 Nov 1;16(11):1302.

49. Lawton M, Baig F, Rolinski M, Ruffman C, Nithi K, May MT, et al. Parkinson's disease subtypes in the oxford Parkinson disease centre (OPDC) discovery cohort. J Parkinsons Dis. 2015;5(2):269–79.

50. PSP Association. STATE OF DIAGNOSIS FOR PROGRESSIVE SUPRANUCLEAR PALSY (PSP) AND CORTICOBASAL DEGENERATION (CBD) REPORT (2023) [Internet]. 2023 [cited 2026 Feb 17]. Available from: https://www.pspassociation.info/wp-content/uploads/2023/06/PSPA_Diagnosis_Report_LR.pdf

AUTHOR STATEMENT

H.R.M. conceptualised the study and developed the study design with the other coinvestigators. R.F., I.C., E.J., T.R., J.B.R., Y.B.S. and M.T.H. contributed to the development of study design and read, edited and approved the final version.

CONFLICTS OF INTEREST

R.G.F. and I.C. are employed by UCL and supported by the National Institute for Health and Care Research and the Medical Research Council. E.J. is supported by Medical Research Council Clinician Scientist (UKRI1388) and Clinical Research Training (MR/S000992/1) fellowships, and grants from the PSP Association (PSPA2023/PROJECTGRANT001) and CurePSP (681-2022/06). J.B.R. is supported by the Medical Research Council (MC_UU_00030/14; MR/T033371/1), the PSP Association, Cambridge Centre for Parkinson-plus, the NIHR Clinical Research Network, and the NIHR Cambridge Biomedical Research Centre (NIHR203312: the views expressed are those of the authors and not necessarily those of the NIHR or the Department of Health and Social Care). Y.B.S. has received paid

For peer review only - http://bmjopen.bmj.com/site/about/guidelines.xhtml

consultancy for Parkinson's UK unrelated to this paper. M.T.H. received research grants from Parkinson's UK, Oxford NIHR BRC, Michael J Fox Foundation, European Platform for Neurodegenerative Disorders and PSP Association. She is an advisory founder, paid consultant and shareholder of KneuHealth Digital Ltd (company number: 14492037), and joint patent holder (18/815,353) to predict dopamine neuron loss in Parkinson's based on simulated dopamine transporter scan (DaTScan) tracer uptake using sensor and clinical data. H.R.M. is employed by UCL. He reports paid consultancy from Arvinas, Aprinoia, Skyhawk, IONIS, AI Therapeutics, Neuron23, LifeArc, Novartis; lecture fees/honoraria - Movement Disorders Society, Bial, Calico and research grants from Parkinson's UK, Cure Parkinson's Trust, PSP Association, Medical Research Council, Michael J Fox Foundation and the NIHR. He is a co-applicant on a patent application related to C9orf72 - Method for diagnosing a neurodegenerative disease (PCT/GB2012/052140).

FIGURE LEGENDS:

Figure 1: Participating sites across England, Scotland and Wales in the ExPRESS study

Figure 2: Study assessments within the ExPRESS study. n refers to the targeted number of recruits by the end of 2030. Dotted lines denote optional procedures.

For peer review only

For peer review only - http://bmjopen.bmj.com/site/about/guidelines.xhtml

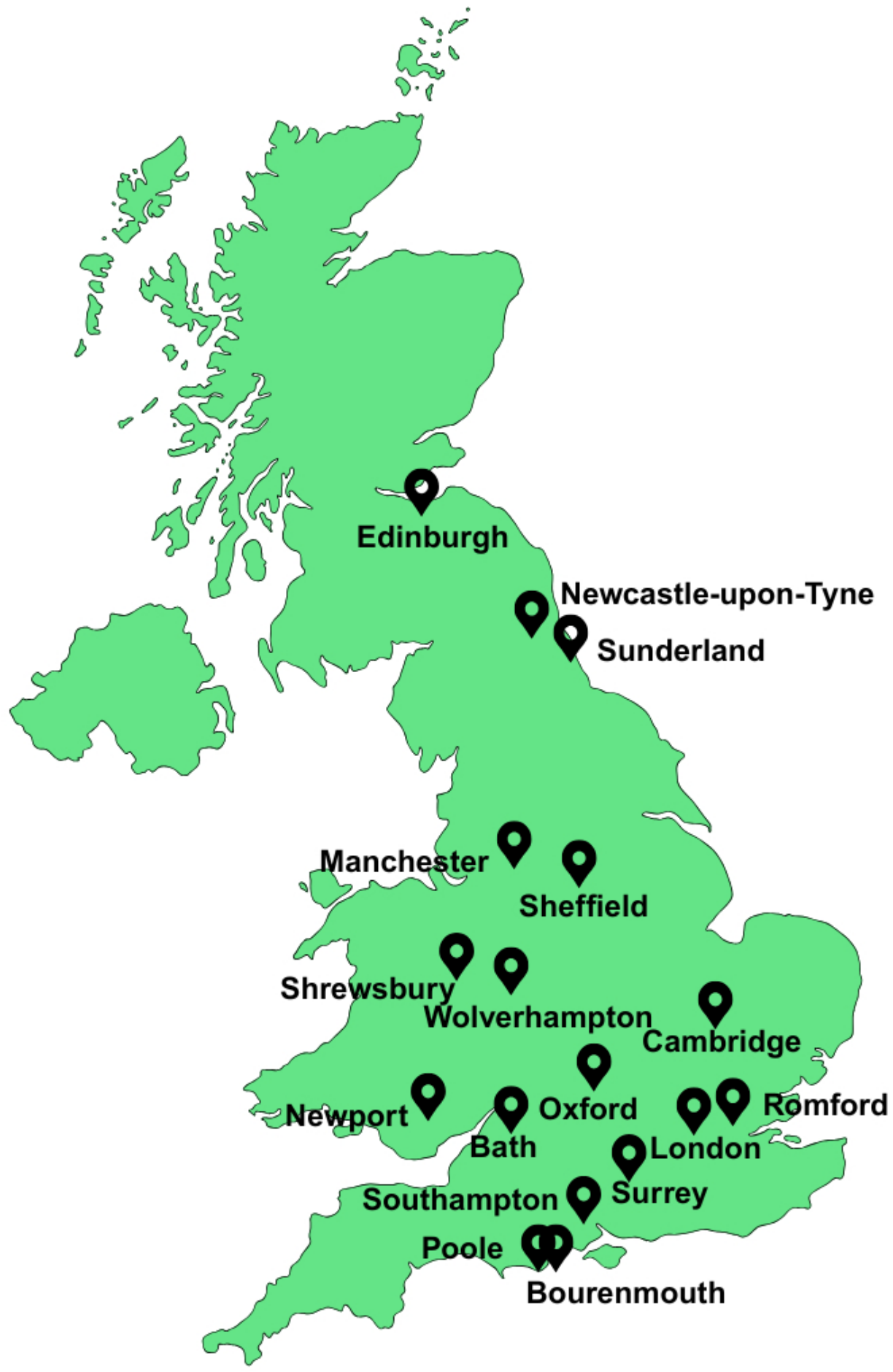

Participating sites across England, Scotland and Wales in the ExPRESS study

285x285mm (96 x 96 DPI)

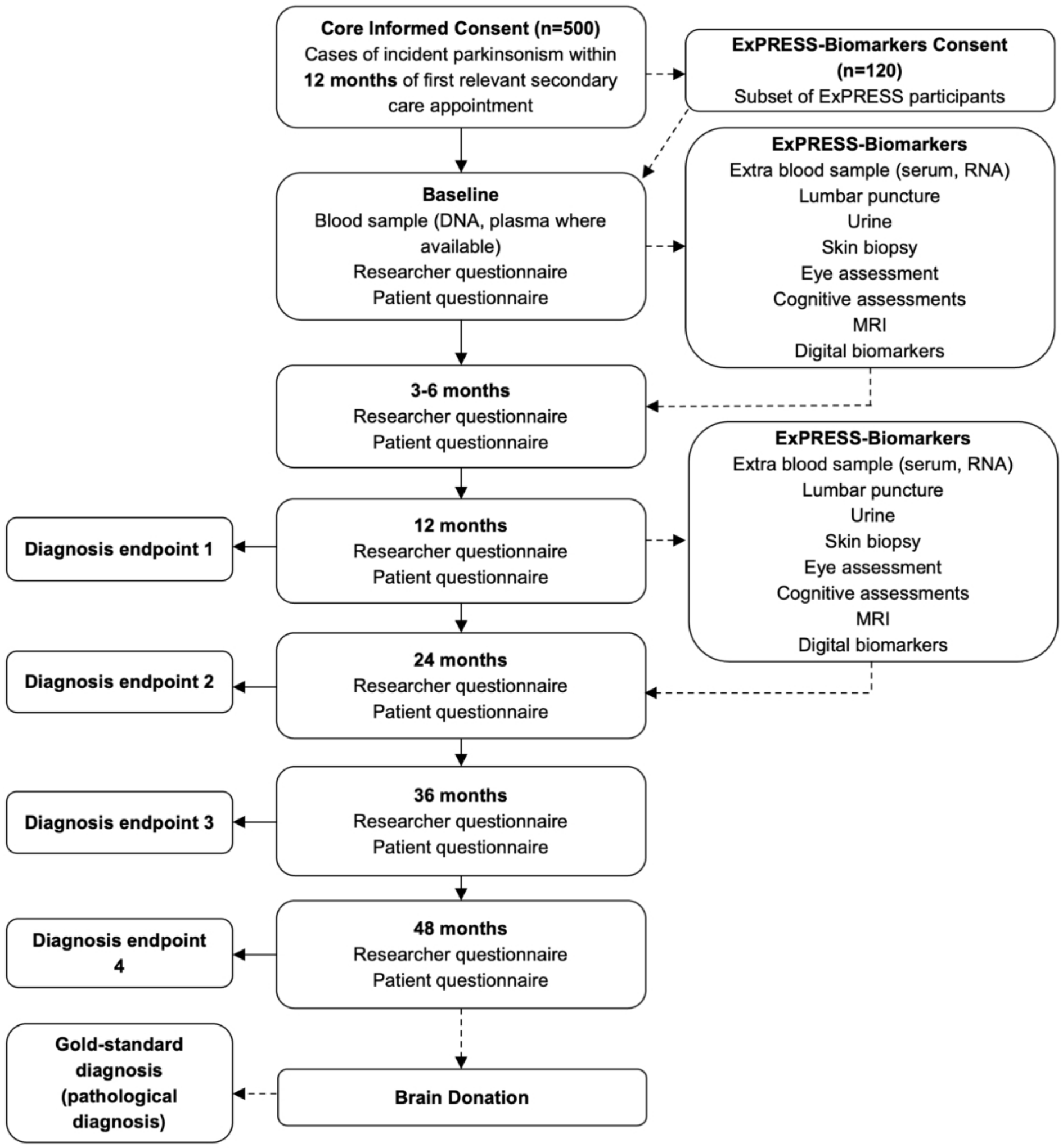

Study assessments within the ExPRESS study. n refers to the targeted number of recruits by the end of 2030. Dotted lines denote optional procedures.

190x206mm (300 x 300 DPI)

BMJ Open

The Early assessment, diagnosis and treatment of Parkinsonism and Related Syndromes study (ExPRESS): a protocol for an observational study on incident parkinsonism

Journal:	*BMJ Open*
Manuscript ID	bmjopen-2026-123017.R1
Article Type:	Protocol
Date Submitted by the Author:	20-Jul-2026
Complete List of Authors:	Fumi, Riona; University College London, Department of Clinical and Movement Neurosciences; University College London, Movement Disorders Centre<br>Caraiscos, Isabella ; University College London, Department of Clinical and Movement Neurosciences; University College London, Movement Disorders Centre<br>Jabbari, Edwin ; University College London, Department of Clinical and Movement Neurosciences; University College London, Movement Disorders Centre<br>Rittman, Timothy; University of Cambridge, Department of Clinical Neurosciences and MRC Cognition and Brain Sciences Unit<br>Rowe, James; University of Cambridge, Department of Clinical Neurosciences and MRC Cognition and Brain Sciences Unit<br>Ben-Shlomo, Yoav; University of Bristol, Population Health Sciences<br>Hu, Michele; University of Oxford, Nuffield Department of Clinical Neurosciences<br>Morris, Huw ; University College London, Department of Clinical and Movement Neurosciences; University College London, Movement Disorders Centre
**Primary Subject Heading**:	Neurology
Secondary Subject Heading:	Epidemiology
Keywords:	Parkinson-s disease < NEUROLOGY, Neurology < INTERNAL MEDICINE, Wearable Devices < Wearable Electronic Devices

SCHOLARONE™
MANUSCRIPTS

The Early assessment, diagnosis and treatment of Parkinsonism and Related Syndromes study (ExPRESS): a protocol for an observational study on incident parkinsonism

Riona G. Fumi[1,2], Isabella Caraiscos[1,2], Edwin Jabbari[1,2], Timothy Rittman[3], James B. Rowe[3], Yoav Ben-Shlomo[4], Michele T. Hu[5] and Huw R. Morris[1,2].

1. Department of Clinical and Movement Neurosciences, UCL Queen Square Institute of Neurology, University College London, London, UK,
2. Movement Disorders Centre, UCL Queen Square Institute of Neurology, London, UK
3. Department of Clinical Neurosciences and MRC Cognition and Brain Sciences Unit, University of Cambridge, UK
4. Population Health Sciences, University of Bristol, Bristol, UK
5. Nuffield Department of Clinical Neurosciences, Oxford University, Oxford, UK

Corresponding author: Riona Giulia Fumi; 33 Queen Square, London, WC1N 3BG; E: riona.fumi@ucl.ac.uk; T: 02031082838.

Author contacts: riona.fumi@ucl.ac.uk, i.caraiscos@ucl.ac.uk, e.jabbari@ucl.ac.uk, tr332@medschl.cam.ac.uk, James.Rowe@mrc-cbu.cam.ac.uk, y.ben-shlomo@bristol.ac.uk, michele.hu@ndcn.ox.ac.uk, h.morris@ucl.ac.uk.

Key words: progressive supranuclear palsy, corticobasal syndrome, multiple system atrophy, Parkinson's disease

Word count: 4529

For peer review only

ABSTRACT

Introduction

Existing evidence from cohort studies of atypical parkinsonism has demonstrated that median time from symptom onset to diagnosis is 3.4 years for progressive supranuclear palsy (PSP) and 3.1 years for multiple system atrophy (MSA). This compares to only 1.0-1.2 years in Parkinson's disease (PD). Earlier diagnosis is essential to facilitate recruitment to disease-modifying treatment trials. In the Early Assessment, Diagnosis and Treatment of Parkinsonism and Related Syndromes study (ExPRESS), we will prospectively assess patients across the UK with incident parkinsonism and determine the best predictors to improve early diagnosis of the "Parkinson-plus" syndromes (PPS) and build a trial-ready cohort.

Methods and analysis

In this longitudinal prospective clinical cohort study, we will recruit 500 people with incident parkinsonism or syndromes associated with later PPS. Participants must be within 12 months of their first secondary care appointment for either motor disturbance or other symptoms of prodromes to PPS. Participants will return a self-completed questionnaire about their symptoms and have a structured neurological assessment by a clinician capturing key clinical features, clinical diagnostic criteria and quantitative clinical scales. Blood for DNA and plasma will be collected at baseline. 120 participants recruited to a subset of study sites will be invited to undergo detailed biomarker assessments such as additional blood collection, lumbar puncture, skin biopsy, magnetic resonance imaging (MRI), cognitive assessments and digital biomarker assessments.

We will stratify participants according to their most likely clinical diagnosis at 4 years post-baseline as the gold standard clinical diagnosis. This will be supplemented by neuropathological examination where available. We will use multivariable logistic modeling to identify early predictors of atypical PPS versus PD. Lifetime follow-up will be carried out using NHS medical records linkage with participant consent.

Ethics and Dissemination

Ethical approvals have been granted by the Queen Square Research Ethics Committee (Ref: 14/LO/1575). with approval from the Health Research Authority and each participating site. Findings from the study will be published in academic journals and presented at UK-wide and international conferences, as well as dissemination via patient advocacy groups and charities including the PSP Association, Cure PSP, Rare Dementia Support, and the MSA Trust.

ARTICLE SUMMARY

Strengths and limitations of this study

- This is a large inception study of parkinsonism, meaning we will recruit participants from a very early disease stage. This will enable collection of multi-modal data relating to early and/or potential pre-diagnostic cases to enable more accurate earlier diagnosis.
- We will recruit participants with both early typical and atypical forms of parkinsonism

For peer review only - http://bmjopen.bmj.com/site/about/guidelines.xhtml

- Participants will be followed-up longitudinally and will extend beyond study visit completion via clinical record linkage. End-point diagnoses will be validated with final clinical diagnosis or pathological follow-up where available.
- Recruitment of early-stage participants from secondary care may pose challenges given clinical care waiting times.

INTRODUCTION

Parkinson's disease (PD) is the most common cause of parkinsonism, with a prevalence of approximately 138 per 100,000 worldwide and an increasing global burden of disease [1]. It is characterised by core symptoms of bradykinesia, rigidity and tremor, which are commonly managed with levodopa medication. However, there are many alternative causes to parkinsonism. These include typical presentations of progressive supranuclear palsy (PSP), corticobasal syndrome (CBS) and Multiple System Atrophy (MSA), some forms of frontotemporal dementia (FTD), and intermediate phenotypes with cognitive and motor features. Approximately half of people presenting with the non-fluent variant primary progressive aphasia type of FTD progress in 3 years to a clinical phenotype resembling PSP or CBS. Together, PSP, CBS and MSA are often known as Parkinson-plus syndromes (PPS), and are clinically and pathologically quite distinct from PD [2].

PSP is caused by a 4-repeat (4R) tauopathy and presents with varying combinations of ocular motor dysfunction, postural instability, akinesia and cognitive dysfunction [3]. Most epidemiological studies of PSP have demonstrated an estimated prevalence of between 3 and 7 per 100,000 based on the classical form of PSP, PSP-Richardson's syndrome, using the National Institute of Neurological Disorders and Stroke (NINDS)-PSP criteria [4–8] Takigawa and colleagues reported a higher estimated prevalence of 17.5 per 100,000 in a large Japanese study [9].

CBS is a clinically and pathologically heterogenous syndrome characterised by asymmetric akinetic-rigidity, dystonia, myoclonus, apraxia, cortico-sensory deficit and alien limb phenomena [10]. Though often caused by the 4R-tauopathy known as corticobasal degeneration (CBD), CBS can also be caused by PSP and Alzheimer's disease (AD) pathologies, as well as by other pathologies such as TDP-43 in rarer cases [11,12]. Its estimated prevalence ranges from 2 and 4 per 100,000 [4–7].

MSA is caused by an oligodendroglial and neuronal alpha-synucleinopathy which has distinct seeding properties to that seen in PD-type Lewy body pathology [13]. Its clinical presentation is primarily characterised by parkinsonism, prominent autonomic dysfunction and/or a cerebellar syndrome [14]. Reported prevalence of MSA ranges from 0.5 and 17 per 100,000 [15,16].

The PPS are often initially misdiagnosed with PD, despite distinctive clinical features (e.g. symmetry, tremor, language, apathy, treatment response, early falls) [17]. In case-control studies of PSP, PD is reported as an initial clinical diagnosis in up to 55% of cases [18–20]. Swallow and Counsell showed that in a combined group of PSP and CBS cases in the PINE study, 53.6% received an initial diagnosis of PD [21]. People who are initially misdiagnosed with

For peer review only - http://bmjopen.bmj.com/site/about/guidelines.xhtml

PD are likely to be re-diagnosed within the first 2 years of a PD diagnosis, as shown by a critical evaluation of the stability of a PD diagnosis across 4 medical institutions in Finland [22]. Here, re-diagnoses from PD included PSP, MSA and clinically undetermined parkinsonism (CUPS). Re-diagnosis from PD to CUPS following expert review heavily suggests that the clinical diagnostic criteria for PD are often not stringently applied in a healthcare setting, which is a crucial contributor to diagnostic delay of the PPS.

Initial misdiagnoses of the PPS are not restricted to PD, and may include other conditions such as dementia with Lewy bodies (DLB), frontotemporal dementia (FTD), AD, Creutzfeldt-Jakob disease (CJD), myelopathy, myasthenia gravis, stroke, functional neurological disorder and pure autonomic failure (PAF) [21,23–25]. Clinico-pathological studies assessing the accuracy of diagnosis of PD, PSP, CBS and MSA have shown that misdiagnoses are common, whereby the positive predictive value of the PPS range between 80 and 90% [26–28]. The most common misdiagnosis of PSP, CBS and MSA is PD, followed by other PPS and other pathologies such as DLB, AD and frontotemporal dementia (FTLD) [27]. A recent clinico-pathological study showed that up to 40% of patients with pathologically confirmed PSP had no parkinsonism at first clinical presentation [29] which may drive the misdiagnosis of early PSP with cognitively presenting disorders.

The initial misdiagnosis of the PPS with PD and other non-PPS syndromes significantly contributes to a lengthy delay between symptom onset and clinical diagnosis. Epidemiological studies have shown that the delay between symptom onset and diagnosis in the PPS ranges from 1.3 years to 3.3 years [20,24,30–32]. In MSA, those with non-motor symptom onset experience a significantly longer delay to clinical diagnosis compared to those with motor onset, with a delay of up to 4.8 ± 2 years in non-motor onset [25]. In the PROSPECT study, a longitudinal UK cohort of atypical parkinsonism, we have shown that median time from symptom onset to diagnosis was 3.4 and 3.1 years respectively, versus an average diagnostic delay of 1.0-1.2 years in PD as per the reported literature [33,34].

Possible reasons for delayed diagnosis include delay to acknowledgement of index symptoms, especially cognitive and language features of early PSP and CBS resembling nonfluent variant Primary Progressive Apraxia (nfvPPA) or FTD, delays in seeking a primary care appointment, lack of awareness of discriminating features (“red flags”) in primary care or even secondary care leading to inaccurate diagnoses of PD, secondary care referral waiting times and poor application of the clinical diagnostic criteria in clinical settings. The Movement Disorders Society (MDS) 2017 diagnostic criteria for oligosymptomatic or prodromal PSP have shown good sensitivity to subsequent Richardson’s syndrome [35]. Although patient-dependent factors such as delays in acknowledgement of symptoms and delays in seeking a healthcare appointment influence diagnostic delay in PSP and CBS, overall diagnostic delay is most impacted after contact with a healthcare professional [30].

Though revisions to the PSP and MSA clinical diagnostic criteria have included ‘suggestive of’ and ‘possible prodromal’ diagnostic categories to identify patients at the earliest disease stages [3,14] diagnostic delay remains a key challenge. It has critical implications on directing patients

For peer review only - http://bmjopen.bmj.com/site/about/guidelines.xhtml

to specialist clinical care as well as referrals to specialist charities such as the PSP Association and The MSA Trust, which help patients and families to plan both logistically and emotionally for progression to death within 5-10 years [36]. Qualitative research by Respondek and colleagues has shown that diagnostic delay plays a large part in the emotionally taxing journey of 'cycling through the healthcare system' [37]. Furthermore, early diagnosis is important for allocation to trials for disease-modifying treatments which have stringent eligibility criteria in line with a therapeutic window.

OBJECTIVES

Primary objective

In the ExPRESS study, we will recruit 500 people with incident parkinsonism and/or related PPS prodromes such as progressive non-fluent aphasia (PNFA) and PAF between October 2024 and July 2030. The primary aim of this study is to identify clinical, genetic, protein, imaging and digital biomarker features associated with early PSP, CBS and MSA within 12 months of a patient's first relevant secondary care appointment. Using a multimodal approach, we aim to identify the best early predictors of PD and the PPS to supplement validated clinical diagnostic criteria.

Secondary objectives

1) To assess the diagnostic delay in PD vs the PPS
2) To understand the diagnostic pathway in PD and the PPS
3) To identify baseline features predictive of re-diagnosis from PD to a PPS
4) To assess how the stringent application of clinical diagnostic criteria for PD, PSP, CBS/CBD, MSA and DLB can improve diagnostic accuracy with and without additional research measures
5) To describe progression to disability in PD vs PPS, and its predictors.

METHODS AND ANALYSIS

ExPRESS Network

The ExPRESS network is a multi-centre UK-wide network currently comprising study sites with clinical or research expertise in parkinsonism. The network is led by Professor Huw Morris (University College London), Professor James Rowe (University of Cambridge), Dr Timothy Rittman (University of Cambridge), Professor Michele Hu (University of Oxford) and Professor Yoav Ben-Shlomo (University of Bristol). There are currently 18 centres across England, Scotland and Wales participating in the ExPRESS study (*Figure 1*). Further sites are being added to increase the geographic and ancestry-based diversity. The ExPRESS study network is embedded within the larger PROSPECT network, which includes participants with atypical parkinsonism of all durations.

Study Funding

For peer review only

The ExPRESS study is jointly funded by the Medical Research Council (MRC) and the National Institute for Health and care Research (NIHR) (MR/Y008219/1) as part of the Rare Disease Research UK initiative (https://rd-research.org.uk/node/express/). The views expressed are those of the authors and not necessarily those of the NIHR or the MRC. The study is also supported by the PSP Association (PSPA 'Understood'), (https://www.pspassociation.org.uk); and the MSA Trust (https://www.msatrust.org.uk).

Patient and Public Involvement

In collaboration with the PSP Association, we consulted patients and carers about study purpose, design, and dissemination plans for the ExPRESS study via support groups and focus groups, which were run both online and in-person. In these sessions, we explained the purpose and outline of the ExPRESS study and invited feedback from both patients and carers. We also consulted the PSP Association Research Involvement Members group about study design. We subsequently designed patient questionnaires to be as concise as possible in line with advice about not making questionnaires too long and time-consuming.

Recruitment

Eligibility criteria state that participants must be within 12 months of their first relevant secondary care appointment for parkinsonism or a related prodrome such as speech and language impairment in nfvPPA (including aphasia and/or PPA) or autonomic dysfunction in PAF. Participants may have been given a pre-existing diagnosis of PD, PSP, CBS, MSA, DLB, indeterminate parkinsonism, a related prodrome or may have no current diagnosis. Participants must be aged 18 or over. Participants with another neurological or psychiatric illness which would significantly impact study participation will be excluded. We will recruit eligible participants from neurology, cognitive or elderly care clinics, or other secondary care services. Participants may self-refer to the study team directly or through patient charities such as Parkinson's UK, the PSP Association or the MSA Trust via ethically approved advertisements on the respective charity websites. We will ask study sites to aim to recruit an approximate minimum of 5 participants per year, however the recruitment of as many participants as possible based on local capacity will be encouraged, and we have not imposed minimum or maximum recruitment numbers.

People with both PD-like or atypical parkinsonism will be recruited to the ExPRESS study. We aim to recruit participants at an approximate 50:50 ratio where approximately half are deemed by the clinician to have a ≥80% likelihood of PD based on medical records and/or referral letters, and half have a <80% likelihood of PD (i.e. likely atypical) to enhance statistical power. We aim to recruit 500 incident parkinsonism cases. Assuming 25% (125) of all participants turn out to have a PPS diagnosis, we will have a 3:1 ratio of PD to PPS and can detect a 0.5 year difference between PD and PPS with 82% power, assuming 5% alpha and after excluding 50 subjects with the longest 10% of delays (80% PPS and 20% PD) which will skew the means. If there are a larger number of PPS cases then our power will be further enhanced.

Study design

For peer review only - http://bmjopen.bmj.com/site/about/guidelines.xhtml

This is a prospective clinical inception cohort study. Study visits will take place at baseline, 6 months, 12 months, 24 months, 36 months and 48 months (see *Figure* 2). At baseline, participants will be asked to donate a blood sample for DNA, and a blood sample for plasma at sites with capacity to process and store samples locally.

Each study site will have delegated clinicians (neurologists, geriatricians or other specialists who are referred patients for autonomic or cognitive disorders) and clinical researchers (research nurses or research assistants) to complete study assessments. At each study visit, a researcher questionnaire will be completed, either following a routine clinical appointment or a during a dedicated research visit. Neurological assessment will be conducted by a clinician and the questionnaire may be completed by the same clinician or by a delegated clinical researcher based off notes from the neurological examination. The following clinical features are captured within the researcher questionnaire at baseline: falls, urinary incontinence, behavioural change, visual hallucinations, REM sleep behaviour disorder, levodopa treatment, levodopa treatment response, intersecting pentagons, verbal fluency in 1 minute, vertical saccade impairment, rigidity, speech impairment, PD and PSP-type bradykinesia, dyspraxia, tremor, resting tremor, jerky movements, dystonia, spasticity, asymmetry, posture, gait freezing, lying or sitting blood pressure and standing or lying blood pressure after 3 minutes. We will ask clinicians to provide the most likely clinical diagnosis at the end of assessment and will be asked to complete checklists for the clinical diagnostic criteria where possible [3,10,14,38,39]. We will also collect clinical diagnostic certainty level and second or differential diagnosis. Further sections of the researcher questionnaire include the Clinical Impression of Severity Index for Parkinson's Disease scale (CISI-PD) and the Schwab and England Activities of Daily Living scale (SEADL).

The researcher questionnaire will be repeated at all follow-up visits. In the follow-up researcher questionnaires, we will ask further questions dedicated to clinical test results, including MRI scan, bladder ultrasound, lumbar puncture, PET scan and caeruloplasmin test results. The MDS-Unified Parkinson’s Disease Rating Scale (UPDRS) Part Ia and the Montreal Cognitive Assessment (MoCA) are also included within the follow-up questionnaires. Should participants be unable to attend an in-person appointment, a video or telephone call will be arranged to collect as much information as possible.

Participants will also be asked to complete questionnaires at each time point. Patient questionnaires will capture basic demographic information, family history, patient history including time and nature of onset, most prominent symptom, asymmetry, key clinical features (falls, urinary dysfunction, visuospatial deficit), levodopa medication and levodopa response. Patients will also be asked to complete the MDS-UPDRS parts Ib and II and the MDS-Cortical Basal Ganglia Functional Scale (MDS-CBFS). Participants will be able to complete questionnaires via an online link, or on paper where they will be posted to the study site.

A subset of 120 participants will be invited to donate extra biomarkers at baseline and 12 months as part of the *ExPRESS-Biomarkers* study at University College London (UCL), King’s College London (KCL), Cambridge, Oxford, Manchester and Sunderland sites. Participants will be invited to donate extra blood samples, cerebrospinal fluid (CSF), urine and a skin biopsy.

For peer review only - http://bmjopen.bmj.com/site/about/guidelines.xhtml

They will also undergo extra assessments such as a cognitive examination comprising the MoCA, Addenbrooke's Cognitive Examination (ACE-III), the Edinburgh Cognitive and Behavioural ALS Screen (ECAS) and a neuropsychometry battery, which have been shown to provide key phenotypic signals in the PPS [40]. Finally, those in the *ExPRESS-Biomarkers* study will have an MRI scan (based on the QMINMC protocol; https://www.rittman.uk/qminmc/), an eye assessment and digital biomarker assessments as developed by the Oxford Parkinson's Disease Centre (OPDC) [41].

The alpha-synuclein seed amplifciation assay (SAA) and high throughput proteomic analysis e.g. NUcleic acid Linked Immuno-Sandwich Assay (NULISA)-CNS-220 will be carried out on plasma and CSF to define biomarkers that can distinguish between PPS and PD in early-stage patients. Further fluid biomarkers will be analysed as they become available.

Digital biomarker assessments will consist of an 8-minute smartphone test performed in clinic assessing gait, balance, tremor, dexterity, reaction time and voice, as developed by the OPDC and previously evaluated across PD, prodromal PD and control participants [42]. Smartphone-based assessments can reliably detect parkinsonism, offering a scalable, objective approach to separating PPS from PD that remains underexplored.

Oculomotor tests will comprise a 10-minute battery, including fixation, horizontal and vertical gaze, smooth pursuit, pro-saccades, anti-saccades, self-paced saccades, and sympathetic/parasympathetic function on pupillometry. Each of these have been shown to be abnormal in some PPS, but have not been compared directly across diagnoses [43,44].

Participants will be asked for consent to view their clinical summary records via the NHS National Care Record Service (NCRS) and will be asked for consent for their GP or consultants to share their medical records, enabling routine remote follow-up beyond study completion.

Storage of clinical data

Study sites will enter collected data into a Research Electronic Data Capture (REDCap), a secure web-based software platform. The database is restricted to authorised study site team members through role-based logins. For MRI scans, a separate imaging platform (XNAT) is used. Digital biomarker data are uploaded directly to the KneuHealth platform (secure cloud infrastructure managed via Amazon Web Services best practices), via the OPDC smartphone application.

Storage of biomaterials

EDTA Blood samples for DNA extraction will be sent to the Rare Inherited Disorders Genomic Laboratory (RIDGL) in London. All other biosamples (plasma, serum, CSF, urine and skin biopsies) will be processed in line with the protocol of the Parkinson's Progression Markers Initiative (PPMI) [45] and stored locally at -70 degrees Celsius. Samples will be transferred to central storage at the Institute of Neurology, UCL, on a periodic basis.

Brain banking

For peer review only - http://bmjopen.bmj.com/site/about/guidelines.xhtml

At the time of consent, participants will be asked whether they would like to receive information on brain donation. Brain banking is not centralised and is performed at the following individual centres: Queen Square Brain Bank, Oxford Brain Bank, Cambridge Brain Bank, Newcastle Brain Tissue Resource or Imperial Brain Bank depending on geographical location. Should participants consent to receiving information, they will be provided with information and consent forms or referred directly to the brain bank by the ExPRESS study team.

At the time of death, confirmation of tissue retrieval will be provided by brain bank coordinators. Post-mortem reports will be shared with the central study team as per the participant's consent to provide pathological verification and allow for clinico-pathological analyses.

Analysis plan

We will aim to identify and validate baseline clinical, genetic, protein, imaging and digital biomarker features which are predictive of final diagnosis. Unless participants are withdrawn before completion of the study, the most recent diagnosis end-point if not pathological diagnosis will be diagnosis end-point 4 (48 months post baseline) (see *Figure 2*). For prediction of final (end-point 4) diagnosis, we will use multivariable logistic regression modelling and decision-tree based machine learning algorithms (such as XGBoost) to identify key clinical features, quantitative scale markers (such as total and sub-score from the MDS-UPDRS, CBFS and MoCA), genetic variants (such as LRRK2, TRIM11, ApoE status and tau haplotype), SAA status and protein biomarker levels (such as neurofilament light, tau and amyloid-beta markers defined from NULISA) collected at baseline which could significantly predict a diagnosis of PD, PSP, CBS or MSA.

With regards to secondary study aims, we will assess the diagnostic delay in PD and each PPS with reference to dates of symptom onset (motor and/or cognitive and/or autonomic) and final diagnosis (end-point 4 diagnosis), and compare diagnostic delay between groups. We will review the diagnostic pathway for each end-point diagnosis by collating data from prospectively collected patient and researcher questionnaires, as well as retrospective review of medical records to extract data preceding study enrolment. For predicting re-diagnosis in patients who are given an initial diagnosis (end-point diagnosis 1) of PD, we will perform similar multivariable logistic regression modelling and decision-tree based machine learning algorithms as described above. We will apply separate models for those in whom the PD diagnosis was retrospectively appropriate or not appropriate (contrasting phenotypic evolution versus misdiagnosis).

Further, we will assess diagnostic accuracy in parkinsonism syndromes using methods as previously described in clinico-pathological research [26]. We will compare pathological diagnosis with each end-point diagnosis and calculate the positive predictive value (PPV) and sensitivity of each clinical diagnosis. We will group cases into the following pathology groups based on primary pathological diagnosis: Lewy Body Disease (LBD), PSP, CBD, AD with clinical presentation of CBS (CBS-AD), MSA and 'other'. We will classify true positives according to the following criteria: LBD pathology with a final clinical diagnosis of PD or DLB, PSP pathology with a final clinical diagnosis of PSP or CBS, CBD pathology with a clinical diagnosis of CBS, AD pathology with a final clinical diagnosis of CBS and MSA pathology with a final clinical diagnosis of MSA.

For peer review only - http://bmjopen.bmj.com/site/about/guidelines.xhtml

We will perform exploratory analysis to assess how application of the clinical diagnostic criteria in the researcher questionnaire impacts diagnostic accuracy, which will be completed dependent on time availability in the research assessment. Finally, we will use cox proportional hazard regression for modelling clinical progression to disability across different disease groups, using a score of <40% in the SEADL or a score of >4 in the CISI-PD disability classification as an indication of disability. We will assess how the emergence of clinical features, change in research scale scores and biomarker levels over time points impact progression to death or disability within each end-point diagnosis group.

For all analyses, data on each clinical feature will be brought together from various different sources. Where both questionnaires have been completed, researcher-reported data will be prioritised over patient-reported data to code clinical features, with the aim of extracting data with the highest accuracy.

The MRI images will be used to assess changes in relation to diagnosis, phenotype, prognosis and progression. These changes will be examined from T1w (regional changes in volumes and thickness), T2w (white matter hyperintensities), diffusion-weighted (tractography and tract-based diffusion metrics) iron-sensitive imaging (nigrosomal and brain stem nuclei/loci) and task-free fMRI (functional connectivity and derived metrics).

We will assess digital smartphone features derived from the 8-minute in clinic assessment and compare these features between different PPS groups to identify whether they can improve the accuracy of early diagnosis. Smartphone-based machine-learning classifiers to (1) detect parkinsonism versus healthy controls and (2) differentiate APS from PD will be developed and evaluated using smartphone feature extraction, SHAP-based feature selection within stratified groups, k-fold cross-validation and trained XGBoost classifiers. Composite scores based on top-performing smartphone features will also be generated to separate PD from APS, and PSP/CBD from MSA cases.

Oculomotor testing will produce quantified parameters from reflexive and volitional saccades, anti-saccades and smooth pursuit including latency, velocity, error rate and gain that will be compared across the APS groups using previously published methods (insert ref 38 and 39 here).

DISCUSSION

This longitudinal prospective clinical cohort study of parkinsonism aims to help with the early differential diagnosis between PD and other degenerative causes of parkinsonism including PPS and FTD/PPA prodromes. Similar prospective longitudinal studies in early PD patients such as the CamPaIGN study [46], PPMI [45], Tracking Parkinson's [47] and OPDC Discovery Cohort study [48] have provided crucial insight into the progression of PD, but have been restricted to the recruitment of participants with a clinical diagnosis of PD, with early cases defined as 3 and 3.5 years from onset in OPDC and Tracking Parkinson's respectively.

For peer review only - http://bmjopen.bmj.com/site/about/guidelines.xhtml

Conversely, epidemiological and convenience cohort studies of PPS and FTD have lacked matched PD cases, such as the PiPPIN study (Coyle Gilchrist 2016, Murley 2020) and the PROSPECT study [17,49]. We propose that by recruiting participants at a 50:50 ratio of ≥80% and <80% likelihood of PD respectively, our cohort will comprise a group of PPS participants with the most relevant disease-control group of typical PD.

We anticipate that the eligibility criteria of 12 months from the first relevant secondary care appointment may pose challenges. Several study sites are tertiary care centres, where patients may have already attended several appointments prior to specialist referral. The predominant participant referral source for the ExPRESS study will be from neurology clinics run by movement disorders consultants and cognitive disorders specialists within the pre-existing PROSPECT network. Patients who attend their first secondary care appointment in a non-neurology clinic may therefore not be identified quickly enough for study recruitment. The high proportion of neurology-centred ExPRESS sites imposes limitations on participant recruitment in those with prodromal features, who are of particular interest and value in identifying truly prodromal cases. This may be offset by the higher index of suspicion for differential diagnoses in specialist centres, and more confident, accurate non-PD diagnoses.

Baseline publications of UK-wide observational parkinsonism cohorts show that a disproportionately high proportion of participants with white European ancestry are typically recruited to Parkinson's research. Cohorts from the PPMI, Tracking Parkinson's and OPDC studies are made up of 95-97% white participants [47,50,51]. To mitigate this risk, we aim to build a representative cohort and have boosted recruitment or initiated site set-up in areas with a high population of people with non-European ancestry, e.g. London North West University Healthcare NHS Trust, King's College Hospital NHS Foundation Trust and the Whittington Health NHS Trust.

The option for remote questionnaire completion and consent to access patient clinical records will serve a critical purpose in allowing data collection remotely and providing an alternative means of assessment should participants become too impaired to visit a study site. Without remote follow-up, drop-out from in-person study visits leads to a bias in less severely impaired disease presentations. It is important to note that this bias is not entirely mitigated by the option for remote follow-up, as completion of full study scales such as the MDS-UPDRS remains a challenge at this stage. The addition of eye movement recording provides a simple non-invasive metric that could ultimately provide a quick readout in clinic. The option for remote testing is intended to improve retention and reduce participant burden, although we recognise the challenges of a "digital divide" in the UK population and sites will seek to mitigate this in discussion with potential participants.

The primary long-term beneficiaries of this study will be patients. Surveys of those with lived experience consistently hear of the demand for an earlier and more accurate diagnosis by quicker referral to specialist care. In a focus group about the ExPRESS study, we asked patients and carers about their perceived benefits of improving early diagnosis. The feedback highlighted that receiving an earlier diagnosis would have reduced anxiety levels and would've

For peer review only - http://bmjopen.bmj.com/site/about/guidelines.xhtml

given families more time to prepare for the future. Additionally, earlier diagnosis facilitates earlier engagement with supporting charities and more options for clinical trials.

We hope that the ExPRESS study will also raise awareness of the differential diagnoses for PD, including PPS, among patients, carers and healthcare professionals, particularly those working in primary care. A survey run by the PSPA in 2023 showed that some patients saw their GP up to 5 times before being referred to a neurologist, representing lack of awareness and contributing to delay to diagnosis [52].

ETHICS AND DISSEMINATION

Ethical approvals have been granted by the Queen Square Research Ethics Committee, as part of the wider PROSPECT study (Ref: 14/LO/1575). Participants will provide informed consent and a study partner will be optionally assigned. A consultee process is in place should participants lack capacity to consent, though we expect the grand majority of participants to have capacity at the specified eligibility window. Capacity will be assessed at each follow-up visit and the study partner will be asked to sign a consultee form should this be required.

Findings from the ExPRESS study will be published in academic journals and presented at UK-wide and international conferences, and communicated by collaborating charities such as the PSP Association, Cure PSP, Rare Dementia Support, and the MSA Trust. Patients, carers and members of the public will be invited to attend meetings and research days showcasing the results of the ExPRESS study, often in collaboration with charity research days. We will continue to involve patients and carers in both study design and results at charity-led support groups.

Data availability statement

The ExPRESS study shares a Data Access Committee (DAC) with that for the overarching PROSPECT study. The DAC aims to encourage scientific collaboration and maximize the impact of data collection to the wider scientific community. A Data Access Application may be made in order to obtain data and/or samples collected as part of the ExPRESS study, by contacting the study coordinator at prospect@ucl.ac.uk.

REFERENCES

1. Luo Y, Qiao L, Li M, Wen X, Zhang W, Li X. Global, regional, national epidemiology and trends of Parkinson's disease from 1990 to 2021: findings from the Global Burden of Disease Study 2021. Front Aging Neurosci. 2024;16:1498756.

2. Prajjwal P, Kolanu ND, Reddy YB, Ahmed A, Marsool MDM, Santoshi K, et al. Association of Parkinson's disease to Parkinson's plus syndromes, Lewy body dementia, and Alzheimer's dementia. Health Sci Rep. 2024 Apr 31;7(4):e2019.

3. Höglinger GU, Respondek G, Stamelou M, Kurz C, Josephs KA, Lang AE, et al. Clinical diagnosis of progressive supranuclear palsy: The movement disorder society criteria. Mov Disord. 2017 June;32(6):853–64.

For peer review only - http://bmjopen.bmj.com/site/about/guidelines.xhtml

4. Swallow DMA, Counsell CE. Prevalence of progressive supranuclear palsy and corticobasal syndrome in Scotland. Neuroepidemiology. 2022 June 2;56(4):291–7.

5. Lyons S, Trépel D, Lynch T, Walsh R, O'Dowd S. The prevalence and incidence of progressive supranuclear palsy and corticobasal syndrome: a systematic review and meta-analysis. J Neurol. 2023 Sept;270(9):4451–65.

6. Swallow DMA, Zheng CS, Counsell CE. Systematic review of prevalence studies of progressive supranuclear palsy and corticobasal syndrome. Mov Disord Clin Pract. 2022 July;9(5):604–13.

7. Coyle-Gilchrist ITS, Dick KM, Patterson K, Vázquez Rodríquez P, Wehmann E, Wilcox A, et al. Prevalence, characteristics, and survival of frontotemporal lobar degeneration syndromes. Neurology. 2016 May 3;86(18):1736–43.

8. Logroscino G, Piccininni M, Graff C, Hardiman O, Ludolph AC, Moreno F, et al. Incidence of Syndromes Associated With Frontotemporal Lobar Degeneration in 9 European Countries. JAMA Neurol. 2023;80(3):279–86.

9. Takigawa H, Kitayama M, Wada-Isoe K, Kowa H, Nakashima K. Prevalence of progressive supranuclear palsy in Yonago: change throughout a decade. Brain Behav. 2016 Dec;6(12):e00557.

10. Armstrong MJ, Litvan I, Lang AE, Bak TH, Bhatia KP, Borroni B, et al. Criteria for the diagnosis of corticobasal degeneration. Neurology. 2013 Jan 29;80(5):496–503.

11. Koga S, Josephs KA, Aiba I, Yoshida M, Dickson DW. Neuropathology and emerging biomarkers in corticobasal syndrome. J Neurol Neurosurg Psychiatry. 2022 June 13;93(9):919–29.

12. Alexander SK, Rittman T, Xuereb JH, Bak TH, Hodges JR, Rowe JB. Validation of the new consensus criteria for the diagnosis of corticobasal degeneration. J Neurol Neurosurg Psychiatry. 2014 Aug;85(8):925–9.

13. Koga S, Sekiya H, Kondru N, Ross OA, Dickson DW. Neuropathology and molecular diagnosis of Synucleinopathies. Mol Neurodegener. 2021 Dec 18;16(1):83.

14. Wenning GK, Stankovic I, Vignatelli L, Fanciulli A, Calandra-Buonaura G, Seppi K, et al. The Movement Disorder Society criteria for the diagnosis of multiple system atrophy. Mov Disord. 2022 June;37(6):1131–48.

15. Kaplan S. Prevalence of multiple system atrophy: A literature review. Rev Neurol (Paris). 2024 May;180(5):438–50.

16. Poewe W, Stankovic I, Halliday G, Meissner WG, Wenning GK, Pellecchia MT, et al. Multiple system atrophy. Nat Rev Dis Primers. 2022 Aug 25;8(1):56.

17. Jabbari E, Holland N, Chelban V, Jones PS, Lamb R, Rawlinson C, et al. Diagnosis across the spectrum of progressive supranuclear palsy and corticobasal syndrome. JAMA Neurol. 2020 Mar 1;77(3):377–87.

For peer review only - http://bmjopen.bmj.com/site/about/guidelines.xhtml

18. Viscidi E, Litvan I, Dam T, Juneja M, Li L, Krzywy H, et al. Clinical Features of Patients With Progressive Supranuclear Palsy in an US Insurance Claims Database. Front Neurol. 2021;12:571800.

19. Kwasny MJ, Oleske DM, Zamudio J, Diegidio R, Höglinger GU. Clinical features observed in general practice associated with the subsequent diagnosis of progressive supranuclear palsy. Front Neurol. 2021 Apr 22;12:637176.

20. Street D, Whiteside D, Rittman T, Rowe JB. Prediagnostic Progressive Supranuclear Palsy - Insights from the UK Biobank. Parkinsonism Relat Disord. 2022;95:59–64.

21. Swallow DMA, Counsell CE. The evolution of diagnosis from symptom onset to death in progressive supranuclear palsy (PSP) and corticobasal degeneration (CBD) compared to Parkinson's disease (PD). J Neurol. 2023 July;270(7):3464–74.

22. Raty V, Kuusimaki T, Majuri J, Vahlberg T, Gardberg M, Noponen T, et al. Stability and Accuracy of a Diagnosis of Parkinson Disease Over 10 Years. Neurology. 2025;104(9):e213499.

23. Shea YF, Shum ACK, Lee SC, Chiu PKC, Leung KS, Kwan YK, et al. Natural clinical course of progressive supranuclear palsy in Chinese patients in Hong Kong. Hong Kong Med J. 2019;25(6):444–52.

24. Morgan JC, Ye X, Mellor JA, Golden KJ, Zamudio J, Chiodo LA, et al. Disease course and treatment patterns in progressive supranuclear palsy: A real-world study. J Neurol Sci. 2021 Feb 15;421(117293):117293.

25. Vichayanrat E, Valerio F, Koay S, De Pablo-Fernandez E, Panicker J, Morris H, et al. Diagnosing premotor multiple system atrophy: Natural history and autonomic testing in an autopsy-confirmed cohort: Natural history and autonomic testing in an autopsy-confirmed cohort. Neurology. 2022 Sept 13;99(11):e1168–77.

26. Hughes AJ, Daniel SE, Ben-Shlomo Y, Lees AJ. The accuracy of diagnosis of parkinsonian syndromes in a specialist movement disorder service. Brain. 2002 Apr;125(Pt 4):861–70.

27. Massey Q, Nihoyannopoulos L, Zeidman P, Warner T, Bhatia K, Gandhi S, et al. Refining the diagnostic accuracy of Parkinsonian disorders using metaphenomic annotation of the clinicopathological literature. NPJ Parkinsons Dis. 2025;11(1):314.

28. Wrigley S, Cullinane PW, Parmera JB, Arca VM, Sifontes Valladares W, Rivera-Sánchez M, et al. Clinical diagnosis of progressive supranuclear palsy (PSP): A clinicopathological comparison of patients with confirmed PSP and clinical mimics. Mov Disord. 2025 Oct;40(10):2116–28.

29. Driver-Dunckley ED, Zhang N, Serrano GE, Dunckley NA, Sue LI, Shill HA, et al. Low clinical sensitivity and unexpectedly high incidence for neuropathologically diagnosed progressive supranuclear palsy. J Neuropathol Exp Neurol. 2023 Apr 20;82(5):438–51.

30. Swallow DMA, Murchie P, Counsell CE. Total Patient Delay: A comparison of patient and clinician/health system delays in the diagnosis of progressive supranuclear palsy and corticobasal syndrome. Mov Disord Clin Pract. 2024 May;11(5):478–87.

For peer review only - http://bmjopen.bmj.com/site/about/guidelines.xhtml

31. Marchand F, Blaise AS, Defebvre L, Cailliau E, Bombois S, Devos D, et al. Clinical prognostic factors in progressive supranuclear palsy: Implications for clinical trials. J Parkinsons Dis. 2024 Nov;14(8):1652–8.

32. Libri I, Altomare D, Bracca V, Rivolta J, Cantoni V, Mattioli I, et al. Time to diagnosis and its predictors in syndromes associated with frontotemporal Lobar Degeneration. Am J Geriatr Psychiatry. 2024 Aug;32(8):1004–13.

33. Breen DP, Evans JR, Farrell K, Brayne C, Barker RA. Determinants of delayed diagnosis in Parkinson's disease. J Neurol. 2013 Aug 10;260(8):1978–81.

34. Cervantes-Arriaga A, Sarabia-Tapia C, Esquivel-Zapata O, López-Alamillo S, Reséndiz-Henriquez E, Corona T, et al. Pitfalls and caveats in the diagnostic pathway of people with ParkinsonÓ?s disease. Revista Mexicana de Neurociencia [Internet]. 2022 Oct 26 [cited 2026 Mar 17];23(4). Available from: http://dx.doi.org/10.24875/RMN.21000041

35. Grimm MJ, Respondek G, Stamelou M, Arzberger T, Ferguson L, Gelpi E, et al. Clinical conditions 'suggestive of Progressive Supranuclear Palsy'-diagnostic performance. Mov Disord. 2020 Dec;35(12):2301–13.

36. Golbe LI. Early diagnosis of progressive supranuclear palsy: bucking the odds. Neurology. 2008 Nov 25;71(22):1754–5.

37. Respondek G, Breslow D, Amirghiasvand C, Ghosh B, Bergmans B, van Wyk L, et al. The lived experiences of people with progressive supranuclear palsy and their caregivers. Neurol Ther. 2023 Feb;12(1):229–47.

38. Postuma RB, Berg D, Stern M, Poewe W, Olanow CW, Oertel W, et al. MDS clinical diagnostic criteria for Parkinson's disease: MDS-PD Clinical Diagnostic Criteria. Mov Disord. 2015 Oct 1;30(12):1591–601.

39. McKeith IG, Boeve BF, Dickson DW, Halliday G, Taylor JP, Weintraub D, et al. Diagnosis and management of dementia with Lewy bodies: Fourth consensus report of the DLB Consortium. Neurology. 2017 July 4;89(1):88–100.

40. Hu MT, Coma AQ, Rowe JB, Zerenner T, Church A, Fumi R, et al. Cognitive and neuropsychiatric profiles distinguish atypical parkinsonian syndromes. Brain. 2025 Sept 3;148(9):3239–51.

41. Arora S, Baig F, Lo C, Barber TR, Lawton MA, Zhan A, et al. Smartphone motor testing to distinguish idiopathic REM sleep behavior disorder, controls, and PD. Neurology. 2018 Oct 16;91(16):e1528–38.

42. Gunter KM, Groenewald K, Aubourg T, Lo C, Welch J, Razzaque J, et al. Smartphone-based prediction of dopaminergic deficit in prodromal and manifest Parkinson's disease. NPJ Digit Med. 2025 Dec 1;8(1):783.

43. Kassavetis P, Kaski D, Anderson T, Hallett M. Eye movement disorders in movement disorders. Mov Disord Clin Pract. 2022 Apr 16;9(3):284–95.

44. Sekar A, Panouillères MTN, Kaski D. Detecting abnormal eye movements in patients with neurodegenerative diseases - current insights. Eye Brain. 2024 Apr 9;16:3–16.

For peer review only - http://bmjopen.bmj.com/site/about/guidelines.xhtml

45. Marek K, Jennings D, Lasch S, Siderowf A, Tanner C, Simuni T, et al. The Parkinson Progression Marker Initiative (PPMI). Prog Neurobiol. 2011 Dec;95(4):629–35.

46. Williams-Gray CH, Mason SL, Evans JR, Foltynie T, Brayne C, Robbins TW, et al. The CamPaIGN study of Parkinson's disease: 10-year outlook in an incident population-based cohort. J Neurol Neurosurg Psychiatry. 2013 Nov;84(11):1258–64.

47. Malek N, Swallow DMA, Grosset KA, Lawton MA, Marrinan SL, Lehn AC, et al. Tracking Parkinson's: Study design and baseline patient data. J Parkinsons Dis. 2015;5(4):947–59.

48. Szewczyk-Krolikowski K, Tomlinson P, Nithi K, Wade-Martins R, Talbot K, Ben-Shlomo Y, et al. The influence of age and gender on motor and non-motor features of early Parkinson's disease: initial findings from the Oxford Parkinson Disease Center (OPDC) discovery cohort. Parkinsonism Relat Disord. 2014 Jan;20(1):99–105.

49. Street D, Jabbari E, Costantini A, Jones PS, Holland N, Rittman T, et al. Progression of atypical parkinsonian syndromes: PROSPECT-M-UK study implications for clinical trials. Brain. 2023 Aug 1;146(8):3232–42.

50. Sfikas E, Koros C. Association of sporadic and genetic Parkinson's disease with cancer: Insights from the PPMI cohort. Genes (Basel). 2025 Nov 1;16(11):1302.

51. Lawton M, Baig F, Rolinski M, Ruffman C, Nithi K, May MT, et al. Parkinson's disease subtypes in the oxford Parkinson disease centre (OPDC) discovery cohort. J Parkinsons Dis. 2015;5(2):269–79.

52. PSP Association. STATE OF DIAGNOSIS FOR PROGRESSIVE SUPRANUCLEAR PALSY (PSP) AND CORTICOBASAL DEGENERATION (CBD) REPORT (2023) [Internet]. 2023 [cited 2026 Feb 17]. Available from: https://www.pspassociation.info/wp-content/uploads/2023/06/PSPA_Diagnosis_Report_LR.pdf

AUTHOR STATEMENT

H.R.M. conceptualised the study and developed the study design with the other coinvestigators. R.F., I.C., E.J., T.R., J.B.R., Y.B.S. and M.T.H. contributed to the development of study design and read, edited and approved the final version. R.F. is the guarantor.

CONFLICTS OF INTEREST

R.G.F. and I.C. are employed by UCL and supported by the National Institute for Health and Care Research and the Medical Research Council. E.J. is supported by Medical Research Council Clinician Scientist (UKRI1388) and Clinical Research Training (MR/S000992/1) fellowships, and grants from the PSP Association (PSPA2023/PROJECTGRANT001) and CurePSP (681-2022/06). J.B.R. is supported by the Medical Research Council (MC_UU_00030/14; MR/T033371/1), the PSP Association, Cambridge Centre for Parkinson-plus, the NIHR Clinical Research Network, and the NIHR Cambridge Biomedical Research Centre (NIHR203312: the views expressed are those of the authors and not necessarily those of the NIHR or the Department of Health and Social Care). Y.B.S. has received paid

For peer review only - http://bmjopen.bmj.com/site/about/guidelines.xhtml

consultancy for Parkinson's UK unrelated to this paper. M.T.H. received research grants from Parkinson's UK, Oxford NIHR BRC, Michael J Fox Foundation, European Platform for Neurodegenerative Disorders and PSP Association. She is an advisory founder, paid consultant and shareholder of KneuHealth Digital Ltd (company number: 14492037), and joint patent holder (18/815,353) to predict dopamine neuron loss in Parkinson's based on simulated dopamine transporter scan (DaTScan) tracer uptake using sensor and clinical data. H.R.M. is employed by UCL. He reports paid consultancy from Arvinas, Aprinoia, Skyhawk, IONIS, AI Therapeutics, Neuron23, LifeArc, Novartis; lecture fees/honoraria - Movement Disorders Society, Bial, Calico and research grants from Parkinson's UK, Cure Parkinson's Trust, PSP Association, Medical Research Council, Michael J Fox Foundation and the NIHR. He is a co-applicant on a patent application related to C9orf72 - Method for diagnosing a neurodegenerative disease (PCT/GB2012/052140). All other authors have no completing interest to declare.

FIGURE LEGENDS:

Figure 1: Participating sites across England, Scotland and Wales in the ExPRESS study

Figure 2: Study assessments within the ExPRESS study. n refers to the targeted number of recruits by the end of 2030. Dotted lines denote optional procedures.

For peer review only

For peer review only - http://bmjopen.bmj.com/site/about/guidelines.xhtml

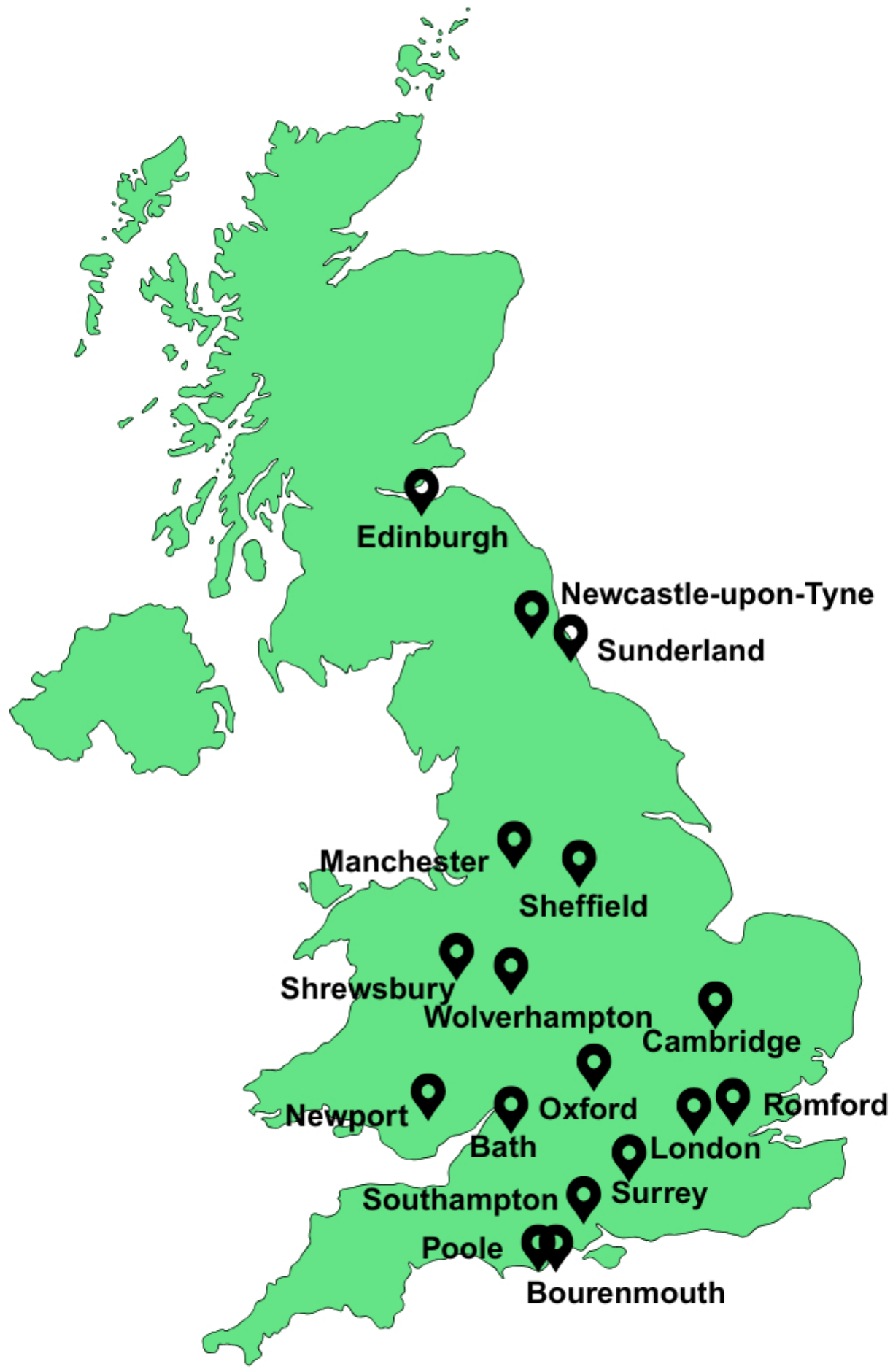

Participating sites across England, Scotland and Wales in the ExPRESS study

285x285mm (96 x 96 DPI)

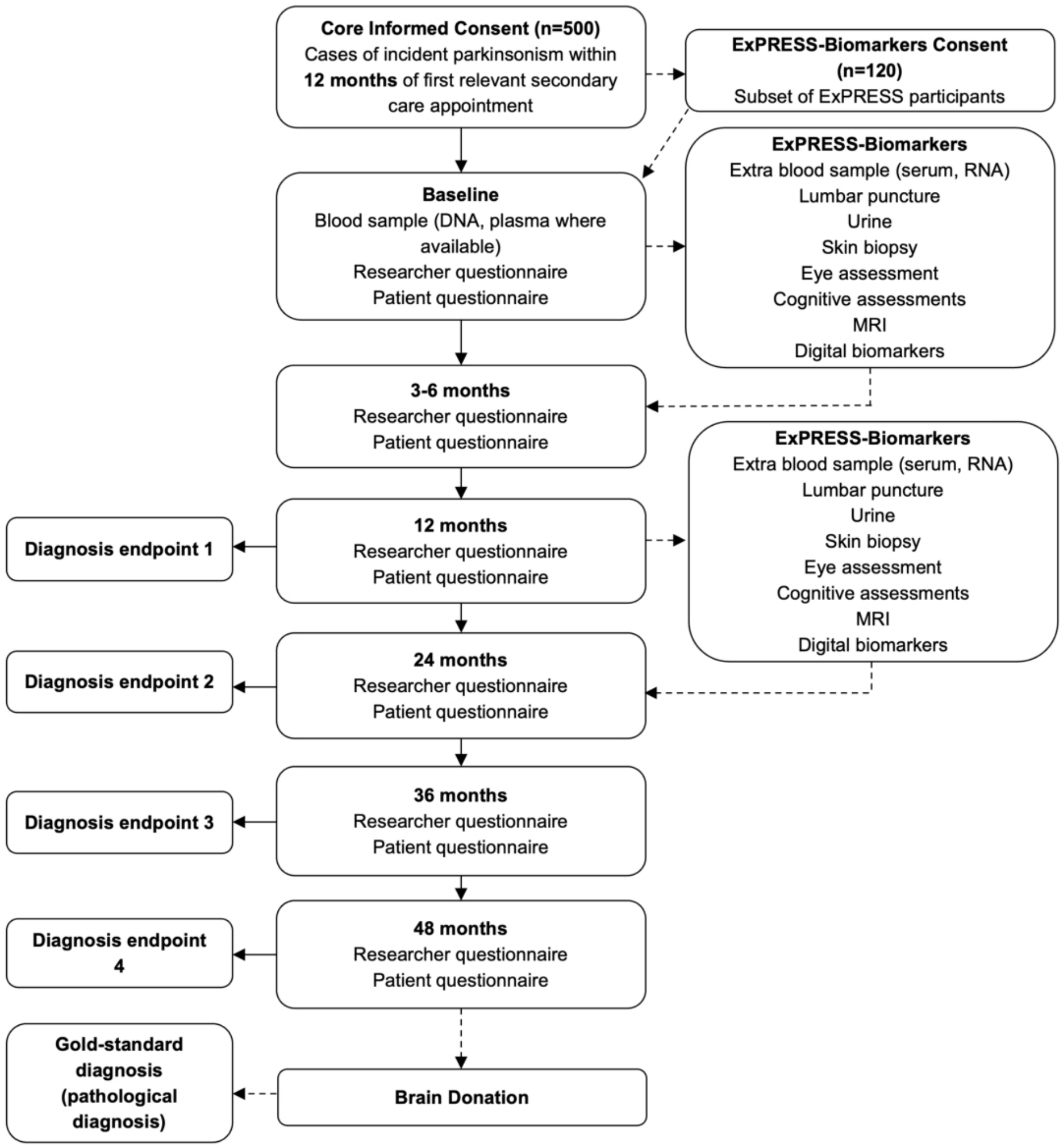

Study assessments within the ExPRESS study. n refers to the targeted number of recruits by the end of 2030. Dotted lines denote optional procedures.

190x206mm (300 x 300 DPI)
