# Supplementary material for: Early assessment, diagnosis and treatment of Parkinsonism and Related Syndromes study (ExPRESS): a protocol for an observational study on incident parkinsonism
Source: BMJ Open. 2026 Jul 29;16(7):e123017. doi: 10.1136/bmjopen-2026-123017 (PMC13479499; doi:10.1136/bmjopen-2026-123017)
Supplement: Supplementary file 2 [file bmjopen-2026-123017.reviewer_comments.pdf]

PEER REVIEW HISTORY

BMJ Open publishes all reviews undertaken for accepted manuscripts. Reviewers are asked to complete a checklist review form and are provided with free text boxes to elaborate on their assessment. These free text comments are reproduced below.

ARTICLE DETAILS

Title (Provisional)

The Early assessment, diagnosis and treatment of Parkinsonism and Related Syndromes study (ExPRESS): a protocol for an observational study on incident parkinsonism

Authors

Fumi, Riona Giulia; Caraiscos, Isabella; Jabbari, Edwin; Rittman, Timothy; Rowe, James B; Ben-Shlomo, Yoav; Hu, Michele T; Morris, Huw R

VERSION 1 - REVIEW

**Reviewer**	**1**
**Name**	**Morris, Meg**
**Affiliation**	**La Trobe Univ, Room 423 HS3**
**Date**	**10-Jun-2026**
**COI**	**None**

This is a novel and clinically relevant protocol for a study that will help to differentiate the diagnosis of “Parkinson-plus” syndromes from idiopathic Parkinson's disease. The manuscript is clearly written and engaging. The manuscript would benefit from further attention to the following points:

1. Abstract. This contains some "local" material which international readers might have difficulty interpreting. For example, please replace the specific words about the "PROSPECT-M-UK study" and replace with a sentence that summarises the point. Many international people will not know about PROSPECT-M-UK.

2. Abstract. In the Abstract the first time you refer to "Parkinson's disease" please call it idiopathic Parkinson's disease to differential it from the other forms of parkinsonism.

3. Abstract. Is this a prospective trial or retrospective data analysis - please specify.

4. Abstract. Please specify who will do the structured neurological assessment - medical practitioner, nurse or physiotherapist?

5. Abstract. Will the subset of 120 people be randomly assigned or will they be the first 120 to volunteer?

6. Abstract. Where you say "We will classify participants ..." do you mean that health professionals or researchers will classify them. Also clarify how this will be done - does somebody just make a subjective clinical diagnosis?

7. Abstract. Specify exactly how long the "long term follow up" will be for.

8. Strengths and Limitations. Not everyone will know what an "inception study" is. Please explain in a few words.

9. Introduction. Please add a reference for the last sentence in the first paragraph.

10. Introduction. Although you discuss the aetiology of PSP, CBS and MMA, very little is said about idiopathic Parkinson's disease. To add balance please give more details about the aetiology and epidemiology of idiopathic PD.

11. Introduction. It is a little hard to follow what you mean by ..."build an algorithm for early prediction of PPS to supplement validated clinical diagnostic criteria'. Please can you unpack this a bit more.

12. Also in the aims you don't mention idiopathic Parkinson's disease - is there a reason for this?

13. Methods: Please justify in detail the sample size justifications - how was the sample size of 500 determined and how many for each PPS variant? Will there also be people with idiopathic PD? Are you sure this sample will be sufficient to answer the research questions?

14. Methods: Where you say "We aim to recruit participants at a 50:50 ratio where approximately half are deemed to have a ≥80% likelihood of PD and half have a <80% likelihood of PD" who will be deeming this and how will they do it?

15. Study design: Please specify the actual design - longitudinal cohort study? Later on you say it is an inception study. The details about study visits should be in the "Procedure" section.

16. Who are "delegated clinicians?"

17. Please add more specific details on how you will assess diagnostic accuracy in parkinsonism syndromes (rather than just referring to another publication).

18. Please clarify what you mean by "harmonised" where you say "researcher-reported data will be harmonised with patient-reported data'.

19. Please rephrase for clarity the following sentence: "We will assess whether digital smartphone features derived from the 8-minute in clinic assessment can improve the accuracy of early diagnosis of APS".

19. Please give more details on consumer / public / patient involvement in the design and implementation of this study.

20. The figures dont specify how many patients will be recruited at each site. Are there minimum numbers?

21. Also how will you ensure that particular clinicians dont dominate the findings by having disproportionally high numbers of their patients included?

VERSION 1 - AUTHOR RESPONSE

1. Abstract. This contains some "local" material which international readers might have difficulty interpreting. For example, please replace the specific words about the "PROSPECT-M-UK study" and replace with a sentence that summarises the point. Many international people will not know about PROSPECT-M-UK.

To broaden the statement, we have replaced 'PROSPECT-M-UK study' with 'cohort studies' within the abstract. Throughout the manuscript, we have also changed "PROSPECT-M-K' to the shortened title, 'PROSPECT', for brevity.

2. Abstract. In the Abstract the first time you refer to "Parkinson's disease" please call it idiopathic Parkinson's disease to differential it from the other forms of parkinsonism.

We have not and will not exclude patients with genetic PD from this study so feel that the broader term PD is more appropriate than idiopathic PD. We had included one instance of using the word 'idiopathic' in the manuscript (line 88), which we have now removed.

3. Abstract. Is this a prospective trial or retrospective data analysis - please specify.

This is an observational study rather than a trial. As we assess participants prospectively we have amended the wording throughout the manuscript to "prospective clinical cohort" to make this clearer.

4. Abstract. Please specify who will do the structured neurological assessment - medical practitioner, nurse or physiotherapist?

The neurological assessment itself will be performed by a team member with expertise in assessing movement disorder patients (they may be a medical practitioner, a nurse, physiotherapist or a clinical researcher). The questionnaire can be filled out by the clinician or retrospectively by research staff based on clinic notes. We have added a sentence to detail this within the methods section on lines 293-299.

5. Abstract. Will the subset of 120 people be randomly assigned or will they be the first 120 to volunteer?

The 'sub-set' refers to participants at 1 of 6 study sites who have been onboarded for the ExPRESS-Biomarkers arm (line 238). At these study sites, all participants will be given the option to take part in additional biosample collection until the target of 120 participants has been met. We have clarified this within the abstract, on line 52.

6. Abstract. Where you say "We will classify participants ..." do you mean that health professionals or researchers will classify them. Also clarify how this will be done - does somebody just make a subjective clinical diagnosis?

At each visit, the most likely clinical diagnosis at each visit will be made by a clinical expert using the clinical diagnostic criteria. However, the use of the word 'classify' here refers to how the data will be used for analyses. The final diagnosis will be used for classifying participants (lines 404-406). In the abstract, we have changed the word 'classify' to 'stratify' to avoid confusion, (line 56).

7. Abstract. Specify exactly how long the "long term follow up" will be for.

The consent form covers long-term follow-up indefinitely so long as the participant has not initiated study withdrawal. We have replaced the term 'long term' with lifetime (line 59).

8. Strengths and Limitations. Not everyone will know what an "inception study" is. Please explain in a few words.

We have defined what we mean by 'inception study' within this section (line 70).

9. Introduction. Please add a reference for the last sentence in the first paragraph.

We have added a reference to introduce the Parkinson Plus Syndromes (PPS) (line 98).

10. Introduction. Although you discuss the aetiology of PSP, CBS and MMA, very little is said about idiopathic Parkinson's disease. To add balance please give more details about the aetiology and epidemiology of idiopathic PD.

To add more balance and emphasise the key differences between PD and the PPS, we have added

a few sentences about PD (line 88-92).

11. Introduction. It is a little hard to follow what you mean by ..."build an algorithm for early prediction of PPS". Please can you unpack this a bit more.

The methodology we will apply has been detailed within the methods section of (lines 406-413), so we have simplified the concept within the objectives section (line 215).

12. Also in the aims you don't mention idiopathic Parkinson's disease - is there a reason for this?

Thank you for pointing this out. We have ensured that PD is included within the aims (line 210-255). Improving the accuracy of diagnosis and improving early diagnosis of the PPS goes hand in hand with improving both in PD.

13. Methods: Please justify in detail the sample size justifications - how was the sample size of 500 determined and how many for each PPS variant? Will there also be people with idiopathic PD? Are you sure this sample will be sufficient to answer the research questions?

Please see lines 277-285 for an explanation of the power calculation and detail on the ratio of a most likely clinical diagnosis of PD versus most likely clinical diagnosis of a PPS. As the PPS are rare and difficult to recruit within the early disease stages due to waiting times for specialist clinical support, we would like to ensure that their recruitment is not limited in any way by setting limits per diagnosis group.

14. Methods: Where you say "We aim to recruit participants at a 50:50 ratio where approximately half are deemed to have a ≥80% likelihood of PD and half have a <80% likelihood of PD" who will be deeming this and how will they do it?

The likelihood of PD will be agreed by the recruiting clinician and the local research professional responsible for recruitment, based off of clinical notes and a referral letter, which we have now specified (lines 279-280). We have added a sentence to detail this, and clarified that the 50:50 ratio is very much an approximate target.

15. Study design: Please specify the actual design - longitudinal cohort study? Later on you say it is an inception study. The details about study visits should be in the "Procedure" section.

This is a longitudinal prospective clinical cohort study with 5 follow-up visits. It is also an inception study as we will recruit early stage and/or newly diagnosed patients. We have amended the wording accordingly throughout the manuscript.

16. Who are "delegated clinicians?"

The clinicians may be neurologists, geriatricians or other specialists who are referred patients for autonomic or cognitive disorders. We have added this detail (lines 294-295).

17. Please add more specific details on how you will assess diagnostic accuracy in parkinsonism syndromes (rather than just referring to another publication).

Thank you, we agree that this should be detailed more thoroughly. We have added in further detail on how clinical diagnostic accuracy will be assessed (lines 428-435).

18. Please clarify what you mean by "harmonised" where you say "researcher-reported data will be harmonised with patient-reported data'.

Data harmonisation in this context refers to bringing in data from multiple different data sources (e.g. for falls, this could be coded from variable fields from the researcher questionnaire and variable fields

in the patient questionnaire). We have amended the paragraph for clarification (lines 448-451).

19. Please rephrase for clarity the following sentence: "We will assess whether digital smartphone features derived from the 8-minute in clinic assessment can improve the accuracy of early diagnosis of APS".

We have re-phrased this sentence (line 460).

20. Please give more details on consumer / public / patient involvement in the design and implementation of this study.

We have added in further detail about how engaging with patients and carers affected study design (lines 255-259) and we have also added in a sentence detailing how patient views helped to highlight the importance of improving early diagnosis(lines 535-542).

21. The figures don't specify how many patients will be recruited at each site. Are there minimum numbers?

We have added in a sentence about recruitment numbers per site (lines 2727-275). Sites will be asked to recruit as many participants as they can based on local capacity.

22. Also how will you ensure that particular clinicians don't dominate the findings by having disproportionally high numbers of their patients included?

As we anticipate there to be some difficulty in the recruitment of high numbers of participants to the ExPRESS study given the aforementioned barriers, we will ask study sites to recruit as many participants as possible and this will remain the priority. This means that sites with a high local capacity for study recruitment may end up with disproportionately high numbers of participants assessed by the same clinician/s. We will run sensitivity analyses including (anonymised) clinician, and should we find evidence of effect modification for clinical data, we will ensure that this is adjusted for and acknowledged.

Further revisions made:

1. *We have used the non-abbreviated terms for NINDS (line 104) and DLB (line 148) which were previously not provided.*
2. *We have given an example of a decision tree machine learning algorithm which we will use (XGBoost) for prediction of final diagnosis (line 409).*
3. *We have added detail on how fluid biosamples will be analysed (lines 348-351, and 411-413).*
